# Genetic polymorphisms identify in species/biovars of *Brucella* isolated in China between 1953 and 2013 by MLST

**DOI:** 10.1186/s12866-018-1149-0

**Published:** 2018-01-10

**Authors:** Dong-ri Piao, Xi Liu, Dong-dong Di, Pei Xiao, Zhong-zhi Zhao, Li-qing Xu, Guo-zhong Tian, Hong-yan Zhao, Wei-xing Fan, Bu-yun Cui, Hai Jiang

**Affiliations:** 1 State Key Laboratory for Infectious Disease Prevention and Control, Collaborative Innovation Center for Diagnosis and Treatment of Infectious Diseases, National Institute for Communicable Disease Control and Prevention, Chinese Center for Disease Control and Prevention, Beijing, China; 2 Laboratory of Zoonoses, China Animal Health and Epidemiology Center, MOA, Qingdao, China; 3 National Institute of Occupational Health and Poison Control, Chinese Center for Disease Control and Prevention, Beijing, China; 4 Qinghai Institute for Endemic Disease Prevention and Control, Xining, China

**Keywords:** *Brucella*, Molecular epidemiology, Genotype, MLST

## Abstract

**Background:**

Brucellosis incidence in China is divided into three stages: high incidence (1950s–1960s), decline (1970s–1980s), and re-emergence (1990s–2010s). At the re-emergence stage, *Brucellosis* incidence grew exponentially and spread to all 32 provinces. We describe the magnitude and the etiological distribution changes in mainland China by genotyping data and emphasize its recent reemergence. We also provide the genetic diversity and molecular epidemiological characteristics of *Brucella*.

**Results:**

From a total of 206 *Brucella* isolates, 19 MLST genotypes (STs) were identified and 13 new STs(ST71–83)were found. MLST grouped the population into three clusters. *B. melitensis*, *B. abortus* and *B. suis* were grouped into cluster 1, 2 and 3 respectively. The predominant genotype in the first cluster by MLST, remained unchanged during the three stages. However, the proportion of genotypes in the three stages had changed. More isolates were clustered in ST8 at the re-emergence stage. STs71–74, which were not found in the two former stages, appeared at the re-emergence stage.

**Conclusions:**

The changing molecular epidemiology of brucellosis improve our understanding of apparent geographic expansion from the historically affected north of China to southern provinces in recent reemergence.

**Electronic supplementary material:**

The online version of this article (10.1186/s12866-018-1149-0) contains supplementary material, which is available to authorized users.

## Background

Human brucellosis remains one of the most common zoonosis that occurs around the world [1] and is endemic in most areas of the world such as the Middle East, Western Asia, Africa, and South America [2]. The prevalence of human brucellosis in China has significantly increased in the past decades. The reported annual human brucellosis incidence showed a fast increasing tendency, from 0.07 per 100, 000 in 1990 to 3.33 per 100, 000 in 2013 [3]. The endemic situation of brucellosis in China has undergone three different stages [4]. It was highly endemic during mid-1950s and 1970s, which subsequently decreased until the mid-1990s, and then markedly increased to date [5]. In the past decades, *Brucella* multiple-locus variable number tandem repeat analysis (MLVA) and multilocus sequencing typing (MLST) had been proposed as a complementary technical approach to classical biotyping methods [6–8], based on its high capacity to identify specific genotypes [9] and establish important epidemiological information, assist in tracing the origin of brucellosis outbreak [10], investigate the genetic relationship within a strain group, and discriminate atypical strains among biovars and species [11].

*B. melitensis* is the predominant species that has been associated with human outbreaks and sporadic brucellosis cases in China, and *B. abortus* and *B. suis* have also been associated with sporadic epidemics [5]. A previous study has shown that the MLST genotype of *Brucella* in Inner Mongolia has undergone significant changes that could be depicted as three stages. In China, The MLST method identified 18 known ST types: ST7,ST8,ST34,ST35 and ST37 (*B. melitensis* biovar 1 and 3), ST1, ST2, ST5, ST28, ST29, ST30, ST31, ST32, ST33 and ST38(*B. abortus* biovar 1 and 3), and ST14, ST17 and ST36 (*B. suis* biovar 1and 3) [3, 12–14]. During the past decades, outbreaks of human brucellosis have been reported in increasing numbers and with an apparent geographic expansion from the historically affected north of Chinato southern provinces [15]. To investigate etiological changes of brucellosis in China, species, biovars and genotypes of Brucella isolates were comprehensively analyzed and compared.

## Methods

### Bacterial strains and DNA preparation

A total of 206 *Brucella* isolates, including 158 *B. melitensis* (118 from human, 21 from sheep, 7 from cattle, 2 from yak, 1 from camel, 1 from deer, 1 from dog, and 7 unknown), 26 *B. abortus* (12 from cattle, 7 from human, 4 from sheep, 2 from yak, and 1 unknown), and 22 *B. suis* (12 from human, 5 from pig, 1 from deer, 1 from goat, 1 from sheep, and 2 unknown), were collected from human and various animals in 25 provinces between 1953 and 2013 (Additional file 1: Table S1). All isolates were selected from the strain base of the culture collection center of the State Key Laboratory for Infectious Disease Prevention and Control, National Institute for Communicable Disease Control and Prevention, Chinese Center for Disease Control and Prevention, which serves as a repository of strains isolated from other Centers for Disease Control and Prevention around the country.

All isolates were cultured to stationary phase at 37 °C in *Brucella* broth. *Brucella* strains were identified as *Brucella* species and biovars according to the traditional biochemical reaction [16], including phage lysis test with Tb, BK2, and R phages, and agglutination with monospecific A and M antisera. Whole genomic DNA was extracted from *Brucella* cultures using a DNeasy Blood and Tissue Kit (Qiagen China Ltd., China) following the manufacturer’s protocol for extraction of genomic DNA from gram-negative bacteria. All sample handling was performed in a BSL-3 biocontainment laboratory at the National Institute for Communicable Disease Control and Prevention, Chinese Center for Disease Control and Prevention(ICDC, China CDC).

### Scheme of MLST

MLST was performed using the method described previously. [7] 9 distinct genomic loci were selected, including seven housekeeping genes, one outer membrane protein gene and one intergenic fragment. PCR cycling parameters were as follows: 95 °C for 5 min, followed by 30 cycles of 94 °C for 30s, 63 °C for 30s, 72 °C for 60 s, and an elongation step at 72 °C for 10 min. The resultant PCR products were purified and sequenced at Shengong Bioscience Company(Shanghai, China). The sequence data were edited using EditSeq module of the Lasergene package (version 7).

### Data analysis of MLST

Each allele of the nine loci was given a distinct numerical designation according to previously published MLST database [6, 7]. Each unique allelic profile for the nine loci was identified as a sequence type (ST).Cluster analysis was performed with UPGMA using the software BioNumerics version 5.1(Applied-Maths, Inc.). A minimum spanning tree (MST) was constructed to determine the minimum evolution path from one strain to all others on the network with a categorical coefficient (with 1/HGDI weight) using the BioNumerics software version 5.1 (Applied-Maths, Inc.). The maps showing the distribution of genotypes in China were drawn with ArcGIS 10.2 for Desktop. Fisher’s exact test was performed with SAS 9.3 [17, 18].

## Results

### Different prominent species and biovars during three incidence stages

As shown in Additional file 1: Table S1, in 1950–1960 s, 11 *B. abortus* (7 biovar 1,1 biovar 2 and 3 biovar 3), 17 *B. melitensis* (6 biovar 1, 7 biovar 2 and 4 biovar 3) and 7 *B. suis* biovar 1 were collected. And in 1970–1980s, 11 *B. abortus* (2 biovar 1, 1 biovar 3,2 biovar 6,1 biovar 7 and 5 biovar 9), 23 *B. melitensis* (15 biovar 1, 5 biovar 2 and 3 biovar 3) and 10 *B. suis* (7 biovar 1, 1 biovar 2 and 2 biovar 3)were collected. While in 1990–2000 s, 4 *B. abortus* (2biovar 1and 2biovar 3), 118 *B. melitensis* (17 biovar 1and 106 biovar 3) and 5 *B. suis* biovar 3were collected. During three incidence stages the most common species was always *B. melitensis*. But, *B. melitensis* biovar 1 was popular before 1990s and *B. melitensis* biovar 3 was predominant in recent re-emergence.

### Genetic diversity of 206 *Brucella* isolates using MLST analysis

ST (9 loci) provided a discriminatory power of 0.565 (Simpson index). Of the 9 loci, *aroA* was the most discriminatory (Simpson index: 0.481) (the Simpson index of all loci ranged from 0.230 to 0.481). (Table 1).

**Table 1:** Polymorphism indexes of ST loci in the 206 *Brucella* isolates

Locus	Diversity index^a^	95% Confidence interval	Number of types	Max (pi)^b^
ST (9 loci)	0.565	0.485–0.644	19	0.651
*aroA*	0.481	0.405–0.558	8	0.703
*omp25*	0.450	0.405–0.558	6	0.722
*cobQ*	0.436	0.364–0.508	4	0.726
*glk*	0.413	0.337–0.489	4	0.726
*gap*	0.406	0.333–0.479	3	0.750
*int-hyp*	0.401	0.331–0.472	3	0.750
*trpE*	0.388	0.323–0.453	4	0.750
*dnaK*	0.308	0.234–0.382	4	0.821
*gyrB*	0.230	0.158–0.303	3	0.873

^a^Simpson index

^b^Fraction of samples with the highest frequency in each particular locus (range: 0.0–1.0)

*B. melitensis*, *B. abortus* and *B. suis* were grouped into cluster 1, 2 and 3 respectively. Nineteen distinct STs were identified among the 9 loci of MLST. Thirteen new STs that were found in the present study were designated as ST71–83. These new STs sequences data were given in Additional file 2: File S2. Among each cluster, the predominant ST in China was ST8 (*n* = 137), ST2 (*n* = 13) and ST17 (*n* = 7), respectively. For ST8, there were extensive hosts, including sheep, goat, cattle, deer, yak, camel and human. For ST2, there were 4 hosts, including cattle, sheep, yak and human. For ST17, there were only 2 hosts, pig and human (Additional file 1: Table S1). In recent re-emergence, there were some changes among each cluster. In cluster 1, some new STs were also found as follows: ST72, ST73, ST74 and ST75. In cluster 2, *B. abortus* biovar was more limited than before. In cluster 3, *B. suis* biovar 3 was predominant and ST17 strains were isolated from Guangxi in1962 as well as 52 years later in Hainan.

There is a good consistency between the genotype identified by MLST and the species determined by traditional typing methods. *B. melitensis* biovar 1 and 3 comprised isolates with genotypes 7, 8, 71, 72, 73, 74, 75, and 81. *B. abortus* biovar 1 and 3 consisted of isolates with genotypes 1, 2, 5, 79, and 82. *B. suis* biovar 1 and 3 included isolates with genotypes 17, 76, 77, 78, 80, and 83 (Table 2 and Additional file 1: Table S1).

**Table 2:** ST distributions during three endemic stages

Cluster	STs	Incidence stage^a^		
		High endemic 1950–1960s	Decline 1970–1980s	Re-emergence 1990–2010s
S1	7	0	1	4
	8	17	20	100
	71	0	2	0
	72	0	0	1
	73	0	0	1
	74	0	0	10
	75	0	0	1
	81	0	1	0
S2	1	3	3	1
	2	5	5	3
	5	3	1	0
	79	1	0	0
	82	0	1	0
S3	17	2	0	5
	76	1	3	0
	77	0	5	0
	78	3	1	0
	80	1	0	0
	83	0	1	0

^a^ The number represents the amount of strains in a particular ST in each respective incidence stage

**Fig. 1:**
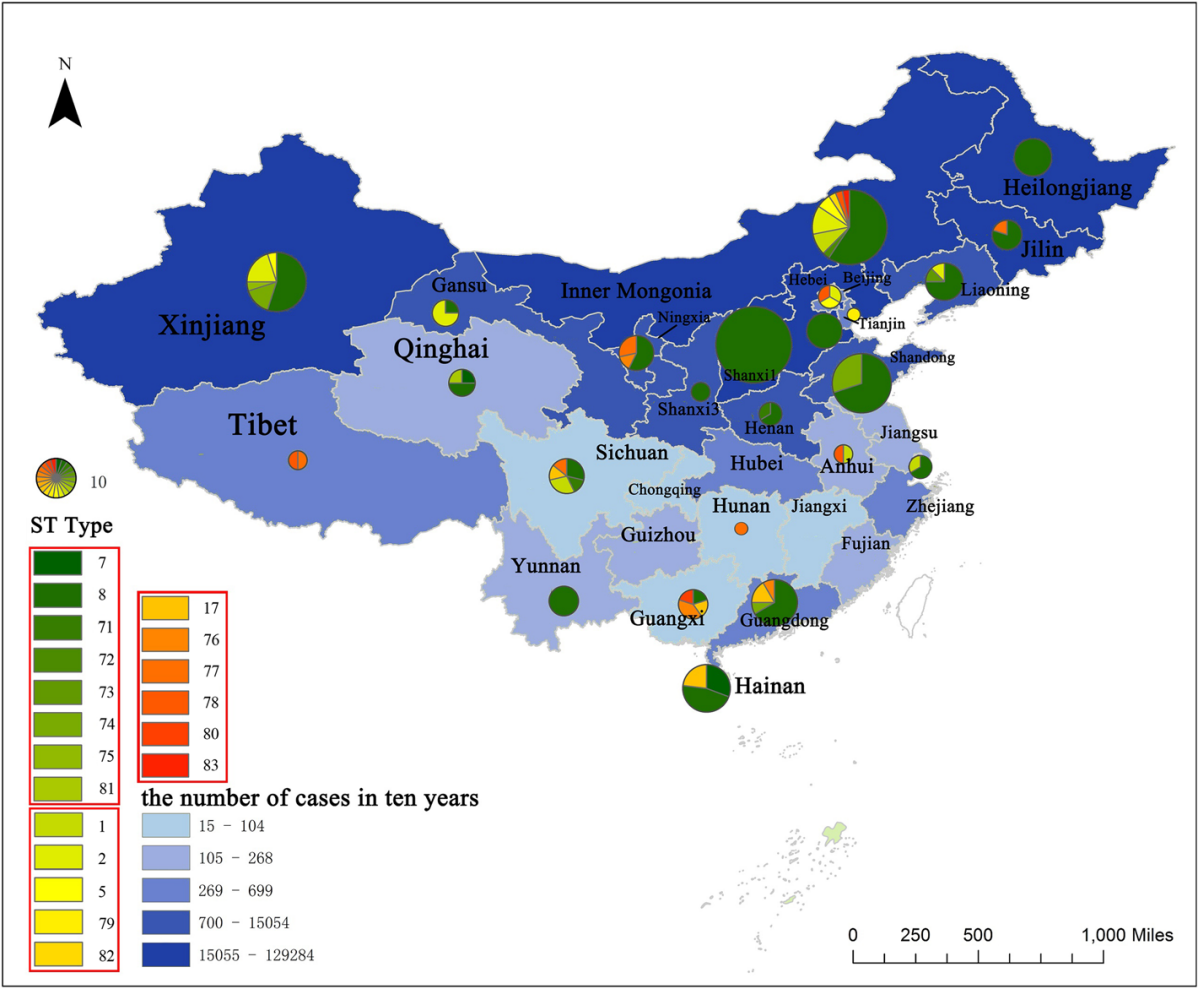
Distribution of MLST in China. Size of circles reflects the number of isolates in a particular province. Color of sectors reflects the genotypes of isolates as the legend displays. The genotypes belong to one identical cluster were framed with red line in the legend. Shade of blocks reflects the number of cases in recent ten years in a particular province

As *B. melitensis* is the main epidemic pathogenic species in China, its isolates were compared to those from provincial level. ST8 was the most widely distributed genotype. Inner Mongolia and Xinjiang remained the most variable provinces based on MLST results. Furthermore, Sichuan, Guangdong, and Guangxi also showed several STs. Genotypic overlaps are presented in Additional file 1: Table S1 (Fig. 1).

### Comparison of STs changes at three incidence stages

MLST analysis showed that in each of the 3 stages, cluster 1, which comprised all *B. melitensis* isolates, remained as the main cluster, whereas relatively fewer strains belonged to clusters 2 and 3(Additional file 3: Figure S1 and Table 2). In addition, a higher number of strains were clustered into cluster 1, particularly in genotype ST8, which was concurrent with an epidemic. ST72–75, which were not detected in the former two stages, were detected at the re-emergence stage (Table 2). The changes in the proportion of isolates in the three clusters among various incidence stages were also statistically significant (*P*   = 3.885E-11, *n*   =   206).

## Discussion

Human brucellosis has reemerged in mainland China since the mid-1990s and has expanded geographically from northern to southern China. Previous epidemiological data showed that *B. melitensis* was predominant species that was associated with outbreaks throughout the country [16]. Results from the present study further implied that different biovars of this species were associated with the two high incidence stages. However, no distinct relationship between biovar and genotyping was observed. A previous study also determined that neither MLVA nor MLST can effectively discriminate *B. melitensis* biovars [19]. These findings indicate that variable number tandem-repeat loci and single-nucleotide polymorphisms, which provide congruent data, might have independently evolved putative genetic determinants in these biovars.

In history, brucellosis was first reported in Inner Mongolia [20] and then eventually spread from the north to the south, and now has now been reported in all 32 Chinese provinces [21]. As seen in both maps, the number of cases in a span of 10 years was highest in the north and gradually declined in the south. Provinces with the highest number of cases, Inner Mongolia and Xinjiang, contained the most variable genotypes that were composed of *B. melitensis*, *B. abortus*, and *B. suis* (Fig. 1). The newly-emerging provinces such Hainan and re-emerging provinces [22] such as Guangdong, also consisted of several genotypes that were even not less than the old epidemic area in the north. The prevention and control of brucellosis in China thus appears to be a very challenging task. *B. melitensis* and *B. suis* were the main causative pathogens of the cases reported in Hainan. Guangxi, which is located adjacent to Hainan, is an important epidemic area of *B. suis* in China. A previous report indicated that Hainan farmers tend to import piglets from Guangxi [23]. In the present study, the strains isolated from Hainan were clustered together with those from Guangxi and showed the same genotype, ST17. Due to insufficiency of epidemiological information, the pigs scattered in the backyard that drink from the same water source as humans, sick pigs, and abortuses might be haphazardly disposed [24]. The importation of infected animals and the limited measure in brucellosis prevention might be the reasons that have caused further spread of brucellosis among pigs and human beings [23]. *B. melitensis* in Hainan, which was isolated in 2013 and genotyped as ST7, were clustered together with the strains from Qinghai that were isolated in 1986, with a similarity of about 90% by MLST. Qinghai province is in the northwest region and Hainan in the very south region of China. The origin of these strains remains unclear. *B. melitensis* and *B. suis* were the main causative pathogens for cases reported in Guangdong. However, these cases were mainly detected in urban areas [25]. Human migration from the north to the south, increased livestock trading and meat production consumption, lack of livestock quarantine measures, and unsafe eating habits [15, 25] might be the main factors that contributed to human infection, without that need for direct contact with livestock.

The appearance of STs 72–74 at the re-emergence stage (isolated in 2011 and 2012 from Inner Mongolia, Liaoning, Guangdong, Shandong, and Xinjiang provinces, which are all border provinces), might have induced the transfer of these 4 STs across the border, thereby resulting in its further spread and suggesting the urgent need to establish inspection and quarantine measures against further spread. At the two high incidence stages, STs were different and more STs (ST8, STs 72–75) were founded at the re-emergence stage. The emergence of new genotypes of *B. melitensis* might be a reason for brucellosis incidence changes in China.

In this study, the phenomenon of host shift was common between *B. melitensis* and *B. abortus*, especially *B. melitensis* (sheep) and the accessory hosts (cattle, deer, yak, and camel). The results have highlighted some of the potential difficulty in national control programs. On the other hand, alterations in socioeconomic and political systems, increasing animal trade and a decreasing awareness by practitioners and public health authorities led to the reemergence of new endemic foci.

This study has some limitations. Due to insufficiencies in reporting brucellosis cases to the local CDC and the low positive rate of culture, there may be discrepancies among collected strains in the culture collection center as well as in its prevalence, thereby causing detection bias. Because brucellosis involves an extensive duration period, a more precise description of the distribution of prevalent genotypes in China should be the focus of future research studies.

## Conclusions

MLST could be used for the epidemiological surveillance of brucellosis. The changing molecular epidemiology of brucellosis improve our understanding of apparent geographic expansion from the historically affected north of China to southern provinces in recent reemergence.

## Additional files

Additional file 1: Table S1. Origins and genotyping results of 206 *Brucella* strains using MLST analysis. (Also see Figure S1 for column information. (XLS 46 kb)

Additional file 2: File S2. New STs (ST71-ST83) sequences data. (DOCX 37 kb)

Additional file 3: Figure S1. UPGMA dendrogram based on the MLST assay showing the similarities of 206 Brucella isolates. Key: serial number for the 206 isolates; Biovar: stains species and biovars by phenotype; Place/Time: the place and time when the strains were collected; Source: the hosts from which the bacteria was isolated; ST: MLST genotype. (PDF 221 kb)

## Abbreviations

CE: Capillary electrophoresis
MST: Minimum spanning tree
STs: MLST genotypes
UPGMA: Unweighted pair group method using arithmetic averages
