# Supplementary material for: Genetic polymorphisms identify in species/biovars of Brucella isolated in China between 1953 and 2013 by MLST
Source: BMC Microbiol. 2018 Jan 10;18:7. doi: 10.1186/s12866-018-1149-0 (PMC5781281; doi:10.1186/s12866-018-1149-0)
Supplement: Supplementary file 2 — New STs (ST71-ST83) sequences data. (DOCX 37 kb) [file 12866_2018_1149_MOESM2_ESM.docx]

ST71

>gap_3

CTCCGCTCCCGCAGACGGTGCCGATCTCACCGTCGTCTATGGTGTCAACAACGACAAGCT

GACGAAGGACCATCTGGTCATCTCCAACGCTTCGTGTACCACCAACTGCCTTGCGCCGGT

GGCTCAGGTTCTCAACGATACTATCGGTATCGAAAAGGGCTTTATGACCACGATCCACTC

CTATACGGGCGACCAGCCGACGCTGGACACCATGCACAAGGATCTCTACCGCGCCCGCGC

CGCTGCCCTTTCCATGATCCCGACCTCGACGGGTGCGGCCAAGGCCGTCGGTCTCGTTCT

GCCGGAACTGAAAGGCAAGCTCTACGGCGTTGCCATTCGCGTCCCGACCCCAAATGTCTC

GGTCGTTGATCTCACCTTCATCGCCAAGCGTGAAACCACCGTTGAAGAAGTCAACAATGC

GATCCGCGAAGCCGCCAATGGCCGCCTCAAGGGCATTCTCGGCTATACCGATGAGAAGCT

CGTCTCGCACGACTTCAACCACGATTCCCATTCCTCGGTCTTCCACACCGACCAGACCAA

GGTTATGGACGGCACCATGGTGCGTATCCTGTCGTGGTACGACAATGAA

>'aroA-16'

TGATCCCTCGTCAACGGCTTTTCCGCTGGTGGCCGCCCTTCTGGTCGAAGGTTCGGAGGTCACCATCCGCAATGTGCTGATGAACCCGACCCGCACCGGCCTGATCCTGACGTTGCAGGAAATGGGGGCGGATATCGAGATCATCGATCCACGCCTTGCCGGCGGCGAGGATGTCGCCGATCTGCGCGTCAAGGCCTCGAAGCTCAAAGGCGTTGTCGTTCCGCCGGAACGTGCGCCTTCGATGATCGATGAATATCCGGTTCTGGCCATTGCCGCGTCTTTTGCGGAAGGCGAAACCGTGATGGACGGTCTCGATGAACTGCGCGTCAAGGAATCGGATCGTCTGGCGGCCGTTGCGCGCGGCCTTGAAGCCAATGGTGTCGATTGTACCGAAGGCGAGATGTCGCTGACGGTTCGTGGCCGCCCCGGCGGCAAGGGGCTGGGCGGTGGCACGGTTGCAACCCACCTCGACCACCGCATCGCGATGAGTTTCCTCGTCATGGGCCTTGCATCGGAAAAGCCGGTTACGGTGGATGACAGCACCATGATCGCCACCTCTTTCCCG

>glk_3

AAACCGGAGGAGGCTGTTGCCACCCGCGTCGTGCTCGGCCCCGGCACGGGCCTTGGCGTG

GCAGGTCTGGTTTGCACACGTCATGCATGGGTTCCGGTTCCCGGTGAAGGCGGTCATATC

GATATCGGTCCACGCACCGAACGCGACTACCAGATTTTCCCGCATATCGAACGCATCGAA

GGGCGTGTCACCGGCGAGCAAATTCTTAGCGGGCGGGGCCTGCGCAACCTCTATCTGGGC

ATCTGCGCGGCCGACAAGATCACGCCCACCCTTGAGACGCCAGTAGACATTACATCCGCC

GGACTGGACGGCAGCAATCCACAAGCCGCAGAAACGCTTGACCTCTTCGCCACCTATCTG

GGGCGGCTTGCGGGCGACCTTGCGCTCATTTTCATGGCGCATGGCGGCGTTTATCTTTCG

GGTGGCATCCCGGTGCGCATCCTTTCCGCCCTCAAGGCCGGTTCGTTCCGCGCAG

>dnaK_2

TTGCCGAGTTCAAGAAGGAAAGTGGCATCGACCTGAAGAACGACAAGCTTGCCCTGCAGC

GCCTCAAGGAAGCTGCCGAAAAGGCCAAGATCGAACTGTCGTCCTCGCAGCAGACCGAAA

TCAACCTGCCGTTCATCACGGCTGACCAGACTGGCCCGAAGCATCTGGCGATCAAGCTGT

CGCGCGCCAAGTTTGAAAGCCTGGTCGATGATCTCGTGCAGCGCACGGTCGAGCCGTGCA

AGGCGGCGCTCAAGGATGCCGGCCTCAAGGCTGGCGAAATTGACGAAGTGGTTCTGGTCG

GCGGCATGACCCGCATGCCCAAGATTCAGGAAGTCGTGAAGGCCTTCTTCGGCAAGGAAC

CGCACAAGGGCGTGAACCCGGATGAAGTCGTGGCCATGGGCGCGGCGATCCAGGGCGGCG

TTTTGCAGGGCGACGTCAAGGACGTGCTGCTGCTCGACGTGACCCCGCTT

>gyrB_1

GGGCACCCTCATCACGGCGCTTGGCACCTCCATCGGCAAGGATGAAACGCACGGCTTCAA

CGCCGACAAGCTGCGTTATCACAAGATCATCATCATGACCGACGCCGACGTCGATGGCGC

CCATATTCGTACGCTTCTGCTCACCTTCTTCTTCCGGCAGATGCCGGAACTGATCGAACG

CGGGCATATCTATATCGCGCAGCCGCCGCTCTATAAGGTGACACGCGGCAAGTCTTCGCA

ATATATCAAGAACGAAGCCGCCTTTGAGGATTTCCTCATCGAAACCGGCCTTGAAGAAAC

GACACTGGAACTGGTGACTGGCGAAATGCGCGCCGGGCCGGATTTGCGCTCGGTGGTGGA

GGATGCGCGCACGCTGCGTCAGCTTCTGCACGGCCTGCACACCCGCTATGACCGCAGCGT

GGTGGAACAGGCGGCAATTGCCGGCCTGCTCAACCCCGATGCCTCAAGG

>trpE_5

ATGCATTTCAATGGCGATATGAATACAGGGCTGACGCTGCGCACCATCCGCATCAAGGAT

GGTGTGGCGGAAATCCGTGCAGGGGCGACGCTTCTGTTCGATTCCAACCCTGACGAGGAA

GAAGCCGAGACCGAATTGAAGGCATCGGCCATGATTGCGGCTGTGCGGGACGCACAGAAG

AGCAATCAGATCGCGGAAGAAAGTGTGGCGGCAAAGGTGGGTGAGGGGGTTTCGATCCTG

CTGGTCGATCACGAGGATTCCTTCGTCCATACGCTTGCCAATTATTTCCGCCAGACGGGC

GCCAAGGTTTCCACCGTGCGTTCACCGGTGGCAGAGGAGATATTCGACCGCGTCAATCCC

GATCTGGTGGTGTTATCGCCGGGACCGGGCTCGCCGCAGGATTTCGATTGCAAGGCGACC

ATCGATAAGGCGCGCAAGCGCCAGCTTCCGATTTTTGGCGTCTGCCTCGGCCTTCAGGCC

CTGGCG

>cobQ_3

CGGCGGATCAGTGACCCGGCGGGTATTGAAGGCAATGTGCGCGATATCGAGGGGCTGGGC

CTTCTCGATATCGAGACGATGACGGAGCCGGAAAAAGTGGTTCGCAATGTTGAGGCGGTG

TCGCTGCTGCATGATGAGCCGCTGGAGGGCTATGAAATCCACATCGGGCGCACCAGCGGG

CCGGATATGGCGCGGCCATTTGCGCGTATCGGCGATCATGATGATGGGGCCGTCTCGCCC

GATGGTCGTATCATGGGAACCTATCTCCACGGTGTTTTCAGTGCGGATCGTTTCCGCCAC

CACTTTTTGCGCGCGCTGGGTGTGGAAGGCGGTCAGATGAATTATCGCGAGAGCGTCGAA

GAGGCTCTGGGCGAACTGGCTGAAGGGCTGGAAGCCTCGCTGGATATTGATGGCCTGTTT

GC

>omp25_8

TCTCGTAATCGTCTCGGCTGCGTTGCTGCCGTTCTCTGCGACCGCTTTTGCTGCCGACGC

CATCCAGGAACAGCCTCCGGTTCCGGCTCCGGTTGAAGTAGCTCCCCAGTATAGCTGGGC

TGGTGGCTATACCGGTCTTTACCTTGGCTACGGCTGGAACAAGGCCAAGACCAGCACCGT

TGGCAGCATCAAGCCTGACGATTGGAAGGCTGGCGCCTTTGCTGGCTGGAACTTCCAGCA

GGACCAGATCGTATACGGCGTTGAAGGTGATGCAGGTTATTCCTGGGCCAAGAAGTCCAA

GGACGGCCTGGAAGTCAAGCAGGGCTTTGAAGGCTCGCTGCGTGCCCGCGTTGGCTACGA

CCTGAACCCGGTTATGCCGTACCTCACGGCTGGTATTGCCGGTTCGCAGATCAAGCTTAA

CAACGGCTTGGACGACGAAAGCAAGTTCCGCGTGGGTTGGACGGCTGGTGCCGGTCTCGA

AGCCAAGCTG

>int_hyp_2

TAACGGTGGTATCATGCCGAGTAAAAGAGCGATCTTTACACCCTTGTCGATCCTGTTTCG

CCCCCGCCACAACACAGCCTGATCGGCAAGCTGTGCTTTGGTGGAGGCGCCGGGTACCGC

CCCCGGGTCCAATGGGTTTATTACACCGTCCGTTTATCACCATAGTCGGCTTGCGCCGAC

AGGACGTATATAGGCGTGGTTTTTACCGATTGGAAGGGGGCTTGTGCGTTTTCGCGCAAG

ACCGACAGAGGTGGTGCGGCCCTTCCGTTCATTTTCCATTGACAGCTTCCGCGCGCTGGT

CAATCCTCACAATATATCGGGATCGGCCTTGAAGAGGCTTGGCGCAGCCGGGGCGGAAAC

CATGGCTGAAACGGGGACGATATGCCCCATTCGAAGGAGAGTGGATATATGAGTGAATAT

CTCGCGGATG

ST72

>gap_3

CTCCGCTCCCGCAGACGGTGCCGATCTCACCGTCGTCTATGGTGTCAACAACGACAAGCT

GACGAAGGACCATCTGGTCATCTCCAACGCTTCGTGTACCACCAACTGCCTTGCGCCGGT

GGCTCAGGTTCTCAACGATACTATCGGTATCGAAAAGGGCTTTATGACCACGATCCACTC

CTATACGGGCGACCAGCCGACGCTGGACACCATGCACAAGGATCTCTACCGCGCCCGCGC

CGCTGCCCTTTCCATGATCCCGACCTCGACGGGTGCGGCCAAGGCCGTCGGTCTCGTTCT

GCCGGAACTGAAAGGCAAGCTCTACGGCGTTGCCATTCGCGTCCCGACCCCAAATGTCTC

GGTCGTTGATCTCACCTTCATCGCCAAGCGTGAAACCACCGTTGAAGAAGTCAACAATGC

GATCCGCGAAGCCGCCAATGGCCGCCTCAAGGGCATTCTCGGCTATACCGATGAGAAGCT

CGTCTCGCACGACTTCAACCACGATTCCCATTCCTCGGTCTTCCACACCGACCAGACCAA

GGTTATGGACGGCACCATGGTGCGTATCCTGTCGTGGTACGACAATGAA

>aroA_6

TGATCCCTCGTCAACGGCTTTTCCGCTGGTGGCCGCCCTTCTGGTCGAAGGTTCGGACGT

CACCATCCGCAATGTGCTGATGAACCCGACCCGCACCGGCCTGATCCTGACGTTGCAGGA

AATGGGGGCGGATATCGAGATCATCGATCCACGCCTTGCCGGCGGCGAGGATGTCGCCGA

TCTGCGCGTCAGGGCCTCGAAGCTGAAAGGCGTTGTCGTTCCGCCGGAACGTGCGCCTTC

GATGATCGATGAATATCCGGTTCTGGCCATTGCCGCGTCTTTTGCGGAAGGCGAAACCGT

GATGGACGGTCTCGATGAACTGCGCGTCAAGGAATCGGATCGTCTGGCGGCCGTTGCGCG

CGGCCTTGAAGCCAATGGTGTCGATTGTACCGAAGGCGAGATGTCGCTGACGGTTCGTGG

CCGCCCCGGCGGCAAGGGGCTGGGCGGTGGCACGGTTGCAACCCACCTCGACCACCGCAT

CGCGATGAGTTTCCTCGTCATGGGCCTTGCATCGGAAAAGCCGGTTACGGTGGATGACAG

CACCATGATCGCCACCTCTTTCCCA

>glk_3

AAACCGGAGGAGGCTGTTGCCACCCGCGTCGTGCTCGGCCCCGGCACGGGCCTTGGCGTG

GCAGGTCTGGTTTGCACACGTCATGCATGGGTTCCGGTTCCCGGTGAAGGCGGTCATATC

GATATCGGTCCACGCACCGAACGCGACTACCAGATTTTCCCGCATATCGAACGCATCGAA

GGGCGTGTCACCGGCGAGCAAATTCTTAGCGGGCGGGGCCTGCGCAACCTCTATCTGGGC

ATCTGCGCGGCCGACAAGATCACGCCCACCCTTGAGACGCCAGTAGACATTACATCCGCC

GGACTGGACGGCAGCAATCCACAAGCCGCAGAAACGCTTGACCTCTTCGCCACCTATCTG

GGGCGGCTTGCGGGCGACCTTGCGCTCATTTTCATGGCGCATGGCGGCGTTTATCTTTCG

GGTGGCATCCCGGTGCGCATCCTTTCCGCCCTCAAGGCCGGTTCGTTCCGCGCAG

>dnaK_2

TTGCCGAGTTCAAGAAGGAAAGTGGCATCGACCTGAAGAACGACAAGCTTGCCCTGCAGC

GCCTCAAGGAAGCTGCCGAAAAGGCCAAGATCGAACTGTCGTCCTCGCAGCAGACCGAAA

TCAACCTGCCGTTCATCACGGCTGACCAGACTGGCCCGAAGCATCTGGCGATCAAGCTGT

CGCGCGCCAAGTTTGAAAGCCTGGTCGATGATCTCGTGCAGCGCACGGTCGAGCCGTGCA

AGGCGGCGCTCAAGGATGCCGGCCTCAAGGCTGGCGAAATTGACGAAGTGGTTCTGGTCG

GCGGCATGACCCGCATGCCCAAGATTCAGGAAGTCGTGAAGGCCTTCTTCGGCAAGGAAC

CGCACAAGGGCGTGAACCCGGATGAAGTCGTGGCCATGGGCGCGGCGATCCAGGGCGGCG

TTTTGCAGGGCGACGTCAAGGACGTGCTGCTGCTCGACGTGACCCCGCTT

>gyrB_1

GGGCACCCTCATCACGGCGCTTGGCACCTCCATCGGCAAGGATGAAACGCACGGCTTCAA

CGCCGACAAGCTGCGTTATCACAAGATCATCATCATGACCGACGCCGACGTCGATGGCGC

CCATATTCGTACGCTTCTGCTCACCTTCTTCTTCCGGCAGATGCCGGAACTGATCGAACG

CGGGCATATCTATATCGCGCAGCCGCCGCTCTATAAGGTGACACGCGGCAAGTCTTCGCA

ATATATCAAGAACGAAGCCGCCTTTGAGGATTTCCTCATCGAAACCGGCCTTGAAGAAAC

GACACTGGAACTGGTGACTGGCGAAATGCGCGCCGGGCCGGATTTGCGCTCGGTGGTGGA

GGATGCGCGCACGCTGCGTCAGCTTCTGCACGGCCTGCACACCCGCTATGACCGCAGCGT

GGTGGAACAGGCGGCAATTGCCGGCCTGCTCAACCCCGATGCCTCAAGG

>trpE_5

ATGCATTTCAATGGCGATATGAATACAGGGCTGACGCTGCGCACCATCCGCATCAAGGAT

GGTGTGGCGGAAATCCGTGCAGGGGCGACGCTTCTGTTCGATTCCAACCCTGACGAGGAA

GAAGCCGAGACCGAATTGAAGGCATCGGCCATGATTGCGGCTGTGCGGGACGCACAGAAG

AGCAATCAGATCGCGGAAGAAAGTGTGGCGGCAAAGGTGGGTGAGGGGGTTTCGATCCTG

CTGGTCGATCACGAGGATTCCTTCGTCCATACGCTTGCCAATTATTTCCGCCAGACGGGC

GCCAAGGTTTCCACCGTGCGTTCACCGGTGGCAGAGGAGATATTCGACCGCGTCAATCCC

GATCTGGTGGTGTTATCGCCGGGACCGGGCTCGCCGCAGGATTTCGATTGCAAGGCGACC

ATCGATAAGGCGCGCAAGCGCCAGCTTCCGATTTTTGGCGTCTGCCTCGGCCTTCAGGCC

CTGGCG

>cobQ_3

CGGCGGATCAGTGACCCGGCGGGTATTGAAGGCAATGTGCGCGATATCGAGGGGCTGGGC

CTTCTCGATATCGAGACGATGACGGAGCCGGAAAAAGTGGTTCGCAATGTTGAGGCGGTG

TCGCTGCTGCATGATGAGCCGCTGGAGGGCTATGAAATCCACATCGGGCGCACCAGCGGG

CCGGATATGGCGCGGCCATTTGCGCGTATCGGCGATCATGATGATGGGGCCGTCTCGCCC

GATGGTCGTATCATGGGAACCTATCTCCACGGTGTTTTCAGTGCGGATCGTTTCCGCCAC

CACTTTTTGCGCGCGCTGGGTGTGGAAGGCGGTCAGATGAATTATCGCGAGAGCGTCGAA

GAGGCTCTGGGCGAACTGGCTGAAGGGCTGGAAGCCTCGCTGGATATTGATGGCCTGTTT

GC

>omp25_8

TCTCGTAATCGTCTCGGCTGCGTTGCTGCCGTTCTCTGCGACCGCTTTTGCTGCCGACGC

CATCCAGGAACAGCCTCCGGTTCCGGCTCCGGTTGAAGTAGCTCCCCAGTATAGCTGGGC

TGGTGGCTATACCGGTCTTTACCTTGGCTACGGCTGGAACAAGGCCAAGACCAGCACCGT

TGGCAGCATCAAGCCTGACGATTGGAAGGCTGGCGCCTTTGCTGGCTGGAACTTCCAGCA

GGACCAGATCGTATACGGCGTTGAAGGTGATGCAGGTTATTCCTGGGCCAAGAAGTCCAA

GGACGGCCTGGAAGTCAAGCAGGGCTTTGAAGGCTCGCTGCGTGCCCGCGTTGGCTACGA

CCTGAACCCGGTTATGCCGTACCTCACGGCTGGTATTGCCGGTTCGCAGATCAAGCTTAA

CAACGGCTTGGACGACGAAAGCAAGTTCCGCGTGGGTTGGACGGCTGGTGCCGGTCTCGA

AGCCAAGCTG

>int_hyp_2

TAACGGTGGTATCATGCCGAGTAAAAGAGCGATCTTTACACCCTTGTCGATCCTGTTTCG

CCCCCGCCACAACACAGCCTGATCGGCAAGCTGTGCTTTGGTGGAGGCGCCGGGTACCGC

CCCCGGGTCCAATGGGTTTATTACACCGTCCGTTTATCACCATAGTCGGCTTGCGCCGAC

AGGACGTATATAGGCGTGGTTTTTACCGATTGGAAGGGGGCTTGTGCGTTTTCGCGCAAG

ACCGACAGAGGTGGTGCGGCCCTTCCGTTCATTTTCCATTGACAGCTTCCGCGCGCTGGT

CAATCCTCACAATATATCGGGATCGGCCTTGAAGAGGCTTGGCGCAGCCGGGGCGGAAAC

CATGGCTGAAACGGGGACGATATGCCCCATTCGAAGGAGAGTGGATATATGAGTGAATAT

CTCGCGGATG

ST73

>gap_3

CTCCGCTCCCGCAGACGGTGCCGATCTCACCGTCGTCTATGGTGTCAACAACGACAAGCT

GACGAAGGACCATCTGGTCATCTCCAACGCTTCGTGTACCACCAACTGCCTTGCGCCGGT

GGCTCAGGTTCTCAACGATACTATCGGTATCGAAAAGGGCTTTATGACCACGATCCACTC

CTATACGGGCGACCAGCCGACGCTGGACACCATGCACAAGGATCTCTACCGCGCCCGCGC

CGCTGCCCTTTCCATGATCCCGACCTCGACGGGTGCGGCCAAGGCCGTCGGTCTCGTTCT

GCCGGAACTGAAAGGCAAGCTCTACGGCGTTGCCATTCGCGTCCCGACCCCAAATGTCTC

GGTCGTTGATCTCACCTTCATCGCCAAGCGTGAAACCACCGTTGAAGAAGTCAACAATGC

GATCCGCGAAGCCGCCAATGGCCGCCTCAAGGGCATTCTCGGCTATACCGATGAGAAGCT

CGTCTCGCACGACTTCAACCACGATTCCCATTCCTCGGTCTTCCACACCGACCAGACCAA

GGTTATGGACGGCACCATGGTGCGTATCCTGTCGTGGTACGACAATGAA

>'aroA-18'

TGATCCCTCGTCAACGGCTTTTCCGCTGGTGGCCGCCCTTCTGGTCGAAGGTTCGGACGTCACCATCCGCAATGTGCTGATGAACCCGACCCGCACCGGCCTGATCCTGACGTTGCAGGAAATGGGGGCGGATATCGAGATCATCGATCCACGCCTTGCCGGCGGCGAGGATGTCGCCGATCTGCGCGTCAAGGCCTCGAAGCTGAAAGGCGTTGTCGTTCCGCCGGAACGTGCGCCTTCGATGATCGATGAATATCCGGTTCTGGCCATTGCCGCGTCTTTTGCGGAAGGCGAAACCGTGATGGACGGTCTCGATGAACTGCGCGTCAAGGAATCGGATCGTCTGGCGGCCGTTGCGCGCGGCCTTGAAGCCAATGGTGTCGATTGTACCGAAGGCGAGATGTCGCTGACGGTTCGTGGCCGCCCCCCCCGCAAGGGGCTGGGGGGGGGCACGGTTGCAACCCACCTCGACCACCGCATCGCGATGAGTTTCCTCGTCATGGGCCTTGCATCGGAAAAGCCGGTTACGGTGGATGACAGCACCATGATCGCC

ACCTCTTTCCCG

>glk_3

AAACCGGAGGAGGCTGTTGCCACCCGCGTCGTGCTCGGCCCCGGCACGGGCCTTGGCGTG

GCAGGTCTGGTTTGCACACGTCATGCATGGGTTCCGGTTCCCGGTGAAGGCGGTCATATC

GATATCGGTCCACGCACCGAACGCGACTACCAGATTTTCCCGCATATCGAACGCATCGAA

GGGCGTGTCACCGGCGAGCAAATTCTTAGCGGGCGGGGCCTGCGCAACCTCTATCTGGGC

ATCTGCGCGGCCGACAAGATCACGCCCACCCTTGAGACGCCAGTAGACATTACATCCGCC

GGACTGGACGGCAGCAATCCACAAGCCGCAGAAACGCTTGACCTCTTCGCCACCTATCTG

GGGCGGCTTGCGGGCGACCTTGCGCTCATTTTCATGGCGCATGGCGGCGTTTATCTTTCG

GGTGGCATCCCGGTGCGCATCCTTTCCGCCCTCAAGGCCGGTTCGTTCCGCGCAG

>dnaK_2

TTGCCGAGTTCAAGAAGGAAAGTGGCATCGACCTGAAGAACGACAAGCTTGCCCTGCAGC

GCCTCAAGGAAGCTGCCGAAAAGGCCAAGATCGAACTGTCGTCCTCGCAGCAGACCGAAA

TCAACCTGCCGTTCATCACGGCTGACCAGACTGGCCCGAAGCATCTGGCGATCAAGCTGT

CGCGCGCCAAGTTTGAAAGCCTGGTCGATGATCTCGTGCAGCGCACGGTCGAGCCGTGCA

AGGCGGCGCTCAAGGATGCCGGCCTCAAGGCTGGCGAAATTGACGAAGTGGTTCTGGTCG

GCGGCATGACCCGCATGCCCAAGATTCAGGAAGTCGTGAAGGCCTTCTTCGGCAAGGAAC

CGCACAAGGGCGTGAACCCGGATGAAGTCGTGGCCATGGGCGCGGCGATCCAGGGCGGCG

TTTTGCAGGGCGACGTCAAGGACGTGCTGCTGCTCGACGTGACCCCGCTT

>gyrB_1

GGGCACCCTCATCACGGCGCTTGGCACCTCCATCGGCAAGGATGAAACGCACGGCTTCAA

CGCCGACAAGCTGCGTTATCACAAGATCATCATCATGACCGACGCCGACGTCGATGGCGC

CCATATTCGTACGCTTCTGCTCACCTTCTTCTTCCGGCAGATGCCGGAACTGATCGAACG

CGGGCATATCTATATCGCGCAGCCGCCGCTCTATAAGGTGACACGCGGCAAGTCTTCGCA

ATATATCAAGAACGAAGCCGCCTTTGAGGATTTCCTCATCGAAACCGGCCTTGAAGAAAC

GACACTGGAACTGGTGACTGGCGAAATGCGCGCCGGGCCGGATTTGCGCTCGGTGGTGGA

GGATGCGCGCACGCTGCGTCAGCTTCTGCACGGCCTGCACACCCGCTATGACCGCAGCGT

GGTGGAACAGGCGGCAATTGCCGGCCTGCTCAACCCCGATGCCTCAAGG

>trpE_5

ATGCATTTCAATGGCGATATGAATACAGGGCTGACGCTGCGCACCATCCGCATCAAGGAT

GGTGTGGCGGAAATCCGTGCAGGGGCGACGCTTCTGTTCGATTCCAACCCTGACGAGGAA

GAAGCCGAGACCGAATTGAAGGCATCGGCCATGATTGCGGCTGTGCGGGACGCACAGAAG

AGCAATCAGATCGCGGAAGAAAGTGTGGCGGCAAAGGTGGGTGAGGGGGTTTCGATCCTG

CTGGTCGATCACGAGGATTCCTTCGTCCATACGCTTGCCAATTATTTCCGCCAGACGGGC

GCCAAGGTTTCCACCGTGCGTTCACCGGTGGCAGAGGAGATATTCGACCGCGTCAATCCC

GATCTGGTGGTGTTATCGCCGGGACCGGGCTCGCCGCAGGATTTCGATTGCAAGGCGACC

ATCGATAAGGCGCGCAAGCGCCAGCTTCCGATTTTTGGCGTCTGCCTCGGCCTTCAGGCC

CTGGCG

>cobQ_3

CGGCGGATCAGTGACCCGGCGGGTATTGAAGGCAATGTGCGCGATATCGAGGGGCTGGGC

CTTCTCGATATCGAGACGATGACGGAGCCGGAAAAAGTGGTTCGCAATGTTGAGGCGGTG

TCGCTGCTGCATGATGAGCCGCTGGAGGGCTATGAAATCCACATCGGGCGCACCAGCGGG

CCGGATATGGCGCGGCCATTTGCGCGTATCGGCGATCATGATGATGGGGCCGTCTCGCCC

GATGGTCGTATCATGGGAACCTATCTCCACGGTGTTTTCAGTGCGGATCGTTTCCGCCAC

CACTTTTTGCGCGCGCTGGGTGTGGAAGGCGGTCAGATGAATTATCGCGAGAGCGTCGAA

GAGGCTCTGGGCGAACTGGCTGAAGGGCTGGAAGCCTCGCTGGATATTGATGGCCTGTTT

GC

>omp25_8

TCTCGTAATCGTCTCGGCTGCGTTGCTGCCGTTCTCTGCGACCGCTTTTGCTGCCGACGC

CATCCAGGAACAGCCTCCGGTTCCGGCTCCGGTTGAAGTAGCTCCCCAGTATAGCTGGGC

TGGTGGCTATACCGGTCTTTACCTTGGCTACGGCTGGAACAAGGCCAAGACCAGCACCGT

TGGCAGCATCAAGCCTGACGATTGGAAGGCTGGCGCCTTTGCTGGCTGGAACTTCCAGCA

GGACCAGATCGTATACGGCGTTGAAGGTGATGCAGGTTATTCCTGGGCCAAGAAGTCCAA

GGACGGCCTGGAAGTCAAGCAGGGCTTTGAAGGCTCGCTGCGTGCCCGCGTTGGCTACGA

CCTGAACCCGGTTATGCCGTACCTCACGGCTGGTATTGCCGGTTCGCAGATCAAGCTTAA

CAACGGCTTGGACGACGAAAGCAAGTTCCGCGTGGGTTGGACGGCTGGTGCCGGTCTCGA

AGCCAAGCTG

>int_hyp_2

TAACGGTGGTATCATGCCGAGTAAAAGAGCGATCTTTACACCCTTGTCGATCCTGTTTCG

CCCCCGCCACAACACAGCCTGATCGGCAAGCTGTGCTTTGGTGGAGGCGCCGGGTACCGC

CCCCGGGTCCAATGGGTTTATTACACCGTCCGTTTATCACCATAGTCGGCTTGCGCCGAC

AGGACGTATATAGGCGTGGTTTTTACCGATTGGAAGGGGGCTTGTGCGTTTTCGCGCAAG

ACCGACAGAGGTGGTGCGGCCCTTCCGTTCATTTTCCATTGACAGCTTCCGCGCGCTGGT

CAATCCTCACAATATATCGGGATCGGCCTTGAAGAGGCTTGGCGCAGCCGGGGCGGAAAC

CATGGCTGAAACGGGGACGATATGCCCCATTCGAAGGAGAGTGGATATATGAGTGAATAT

CTCGCGGATG

ST74

>gap_3

CTCCGCTCCCGCAGACGGTGCCGATCTCACCGTCGTCTATGGTGTCAACAACGACAAGCT

GACGAAGGACCATCTGGTCATCTCCAACGCTTCGTGTACCACCAACTGCCTTGCGCCGGT

GGCTCAGGTTCTCAACGATACTATCGGTATCGAAAAGGGCTTTATGACCACGATCCACTC

CTATACGGGCGACCAGCCGACGCTGGACACCATGCACAAGGATCTCTACCGCGCCCGCGC

CGCTGCCCTTTCCATGATCCCGACCTCGACGGGTGCGGCCAAGGCCGTCGGTCTCGTTCT

GCCGGAACTGAAAGGCAAGCTCTACGGCGTTGCCATTCGCGTCCCGACCCCAAATGTCTC

GGTCGTTGATCTCACCTTCATCGCCAAGCGTGAAACCACCGTTGAAGAAGTCAACAATGC

GATCCGCGAAGCCGCCAATGGCCGCCTCAAGGGCATTCTCGGCTATACCGATGAGAAGCT

CGTCTCGCACGACTTCAACCACGATTCCCATTCCTCGGTCTTCCACACCGACCAGACCAA

GGTTATGGACGGCACCATGGTGCGTATCCTGTCGTGGTACGACAATGAA

>aroA_2

TGATCCCTCGTCAACGGCTTTTCCGCTGGTGGCCGCCCTTCTGGTCGAAGGTTCGGACGT

CACCATCCGCAATGTGCTGATGAACCCGACCCGCACCGGCCTGATCCTGACGTTGCAGGA

AATGGGGGCGGATATCGAGATCATCGATCCACGCCTTGCCGGCGGCGAGGATGTCGCCGA

TCTGCGCGTCAAGGCCTCGAAGCTGAAAGGCGTTGTCGTTCCGCCGGAACGTGCGCCTTC

GATGATCGATGAATATCCGGTTCTGGCCATTGCCGCGTCTTTTGCGGAAGGCGAAACCGT

GATGGACGGTCTCGATGAACTGCGCGTCAAGGAATCGGATCGTCTGGCGGCCGTTGCGCG

CGGCCTTGAAGCCAATGGTGTCGATTGTACCGAAGGCGAGATGTCGCTGACGGTTCGTGG

CCGCCCCGGCGGCAAGGGGCTGGGCGGTGGCACGGTTGCAACCCACCTCGACCACCGCAT

CGCGATGAGTTTCCTCGTCATGGGCCTTGCATCGGAAAAGCCGGTTACGGTGGATGACAG

CACCATGATCGCCACCTCTTTCCCG

>glk_3

AAACCGGAGGAGGCTGTTGCCACCCGCGTCGTGCTCGGCCCCGGCACGGGCCTTGGCGTG

GCAGGTCTGGTTTGCACACGTCATGCATGGGTTCCGGTTCCCGGTGAAGGCGGTCATATC

GATATCGGTCCACGCACCGAACGCGACTACCAGATTTTCCCGCATATCGAACGCATCGAA

GGGCGTGTCACCGGCGAGCAAATTCTTAGCGGGCGGGGCCTGCGCAACCTCTATCTGGGC

ATCTGCGCGGCCGACAAGATCACGCCCACCCTTGAGACGCCAGTAGACATTACATCCGCC

GGACTGGACGGCAGCAATCCACAAGCCGCAGAAACGCTTGACCTCTTCGCCACCTATCTG

GGGCGGCTTGCGGGCGACCTTGCGCTCATTTTCATGGCGCATGGCGGCGTTTATCTTTCG

GGTGGCATCCCGGTGCGCATCCTTTCCGCCCTCAAGGCCGGTTCGTTCCGCGCAG

>'dnak-12'

TTGCCGAGTTCAAGAAGGAAAGTGGCATCGACTTGAAGAACGACAAGCTTGCCCTGCAGCGCCTCAAGGAAGCTGCCGAAAAGGCCAAGATCGAACTGTCGTCCTCGCAGCAGACCGAAATCAACCTGCCGTTCATCACGGCTGACCAGACTGGCCCGAAGCATCTGGCGATCAAGCTGTCGCGCGCCAAGTTTGAAAGCCTGGTCGATGATCTCGTGCAGCGCACGGTCGAGCCGTGCAAGGCGGCGCTCAAGGATGCCGGCCTCAAGGCTGGCGAAATTGACGAAGTGGTTCTGGTCGGCGGCATGACCCGCATGCCCAAGATTCAGGAAGTCGTGAAGGCCTTCTTCGGCAAGGAACCGCACAAGGGCGTGAACCCGGATGAAGTCGTGGCCATGGGCGCGGCGATCCAGGGCGGCGTTTTGCAGGGCGACGTCAAGGACGTGCTGCTGCTCGACGTGACCCCGCTT

>gyrB_1

GGGCACCCTCATCACGGCGCTTGGCACCTCCATCGGCAAGGATGAAACGCACGGCTTCAA

CGCCGACAAGCTGCGTTATCACAAGATCATCATCATGACCGACGCCGACGTCGATGGCGC

CCATATTCGTACGCTTCTGCTCACCTTCTTCTTCCGGCAGATGCCGGAACTGATCGAACG

CGGGCATATCTATATCGCGCAGCCGCCGCTCTATAAGGTGACACGCGGCAAGTCTTCGCA

ATATATCAAGAACGAAGCCGCCTTTGAGGATTTCCTCATCGAAACCGGCCTTGAAGAAAC

GACACTGGAACTGGTGACTGGCGAAATGCGCGCCGGGCCGGATTTGCGCTCGGTGGTGGA

GGATGCGCGCACGCTGCGTCAGCTTCTGCACGGCCTGCACACCCGCTATGACCGCAGCGT

GGTGGAACAGGCGGCAATTGCCGGCCTGCTCAACCCCGATGCCTCAAGG

>trpE_5

ATGCATTTCAATGGCGATATGAATACAGGGCTGACGCTGCGCACCATCCGCATCAAGGAT

GGTGTGGCGGAAATCCGTGCAGGGGCGACGCTTCTGTTCGATTCCAACCCTGACGAGGAA

GAAGCCGAGACCGAATTGAAGGCATCGGCCATGATTGCGGCTGTGCGGGACGCACAGAAG

AGCAATCAGATCGCGGAAGAAAGTGTGGCGGCAAAGGTGGGTGAGGGGGTTTCGATCCTG

CTGGTCGATCACGAGGATTCCTTCGTCCATACGCTTGCCAATTATTTCCGCCAGACGGGC

GCCAAGGTTTCCACCGTGCGTTCACCGGTGGCAGAGGAGATATTCGACCGCGTCAATCCC

GATCTGGTGGTGTTATCGCCGGGACCGGGCTCGCCGCAGGATTTCGATTGCAAGGCGACC

ATCGATAAGGCGCGCAAGCGCCAGCTTCCGATTTTTGGCGTCTGCCTCGGCCTTCAGGCC

CTGGCG

>cobQ_3

CGGCGGATCAGTGACCCGGCGGGTATTGAAGGCAATGTGCGCGATATCGAGGGGCTGGGC

CTTCTCGATATCGAGACGATGACGGAGCCGGAAAAAGTGGTTCGCAATGTTGAGGCGGTG

TCGCTGCTGCATGATGAGCCGCTGGAGGGCTATGAAATCCACATCGGGCGCACCAGCGGG

CCGGATATGGCGCGGCCATTTGCGCGTATCGGCGATCATGATGATGGGGCCGTCTCGCCC

GATGGTCGTATCATGGGAACCTATCTCCACGGTGTTTTCAGTGCGGATCGTTTCCGCCAC

CACTTTTTGCGCGCGCTGGGTGTGGAAGGCGGTCAGATGAATTATCGCGAGAGCGTCGAA

GAGGCTCTGGGCGAACTGGCTGAAGGGCTGGAAGCCTCGCTGGATATTGATGGCCTGTTT

GC

>omp25_8

TCTCGTAATCGTCTCGGCTGCGTTGCTGCCGTTCTCTGCGACCGCTTTTGCTGCCGACGC

CATCCAGGAACAGCCTCCGGTTCCGGCTCCGGTTGAAGTAGCTCCCCAGTATAGCTGGGC

TGGTGGCTATACCGGTCTTTACCTTGGCTACGGCTGGAACAAGGCCAAGACCAGCACCGT

TGGCAGCATCAAGCCTGACGATTGGAAGGCTGGCGCCTTTGCTGGCTGGAACTTCCAGCA

GGACCAGATCGTATACGGCGTTGAAGGTGATGCAGGTTATTCCTGGGCCAAGAAGTCCAA

GGACGGCCTGGAAGTCAAGCAGGGCTTTGAAGGCTCGCTGCGTGCCCGCGTTGGCTACGA

CCTGAACCCGGTTATGCCGTACCTCACGGCTGGTATTGCCGGTTCGCAGATCAAGCTTAA

CAACGGCTTGGACGACGAAAGCAAGTTCCGCGTGGGTTGGACGGCTGGTGCCGGTCTCGA

AGCCAAGCTG

>int_hyp_2

TAACGGTGGTATCATGCCGAGTAAAAGAGCGATCTTTACACCCTTGTCGATCCTGTTTCG

CCCCCGCCACAACACAGCCTGATCGGCAAGCTGTGCTTTGGTGGAGGCGCCGGGTACCGC

CCCCGGGTCCAATGGGTTTATTACACCGTCCGTTTATCACCATAGTCGGCTTGCGCCGAC

AGGACGTATATAGGCGTGGTTTTTACCGATTGGAAGGGGGCTTGTGCGTTTTCGCGCAAG

ACCGACAGAGGTGGTGCGGCCCTTCCGTTCATTTTCCATTGACAGCTTCCGCGCGCTGGT

CAATCCTCACAATATATCGGGATCGGCCTTGAAGAGGCTTGGCGCAGCCGGGGCGGAAAC

CATGGCTGAAACGGGGACGATATGCCCCATTCGAAGGAGAGTGGATATATGAGTGAATAT

CTCGCGGATG

ST75

>gap_3

CTCCGCTCCCGCAGACGGTGCCGATCTCACCGTCGTCTATGGTGTCAACAACGACAAGCT

GACGAAGGACCATCTGGTCATCTCCAACGCTTCGTGTACCACCAACTGCCTTGCGCCGGT

GGCTCAGGTTCTCAACGATACTATCGGTATCGAAAAGGGCTTTATGACCACGATCCACTC

CTATACGGGCGACCAGCCGACGCTGGACACCATGCACAAGGATCTCTACCGCGCCCGCGC

CGCTGCCCTTTCCATGATCCCGACCTCGACGGGTGCGGCCAAGGCCGTCGGTCTCGTTCT

GCCGGAACTGAAAGGCAAGCTCTACGGCGTTGCCATTCGCGTCCCGACCCCAAATGTCTC

GGTCGTTGATCTCACCTTCATCGCCAAGCGTGAAACCACCGTTGAAGAAGTCAACAATGC

GATCCGCGAAGCCGCCAATGGCCGCCTCAAGGGCATTCTCGGCTATACCGATGAGAAGCT

CGTCTCGCACGACTTCAACCACGATTCCCATTCCTCGGTCTTCCACACCGACCAGACCAA

GGTTATGGACGGCACCATGGTGCGTATCCTGTCGTGGTACGACAATGAA

>aroA_2

TGATCCCTCGTCAACGGCTTTTCCGCTGGTGGCCGCCCTTCTGGTCGAAGGTTCGGACGT

CACCATCCGCAATGTGCTGATGAACCCGACCCGCACCGGCCTGATCCTGACGTTGCAGGA

AATGGGGGCGGATATCGAGATCATCGATCCACGCCTTGCCGGCGGCGAGGATGTCGCCGA

TCTGCGCGTCAAGGCCTCGAAGCTGAAAGGCGTTGTCGTTCCGCCGGAACGTGCGCCTTC

GATGATCGATGAATATCCGGTTCTGGCCATTGCCGCGTCTTTTGCGGAAGGCGAAACCGT

GATGGACGGTCTCGATGAACTGCGCGTCAAGGAATCGGATCGTCTGGCGGCCGTTGCGCG

CGGCCTTGAAGCCAATGGTGTCGATTGTACCGAAGGCGAGATGTCGCTGACGGTTCGTGG

CCGCCCCGGCGGCAAGGGGCTGGGCGGTGGCACGGTTGCAACCCACCTCGACCACCGCAT

CGCGATGAGTTTCCTCGTCATGGGCCTTGCATCGGAAAAGCCGGTTACGGTGGATGACAG

CACCATGATCGCCACCTCTTTCCCG

>glk_3

AAACCGGAGGAGGCTGTTGCCACCCGCGTCGTGCTCGGCCCCGGCACGGGCCTTGGCGTG

GCAGGTCTGGTTTGCACACGTCATGCATGGGTTCCGGTTCCCGGTGAAGGCGGTCATATC

GATATCGGTCCACGCACCGAACGCGACTACCAGATTTTCCCGCATATCGAACGCATCGAA

GGGCGTGTCACCGGCGAGCAAATTCTTAGCGGGCGGGGCCTGCGCAACCTCTATCTGGGC

ATCTGCGCGGCCGACAAGATCACGCCCACCCTTGAGACGCCAGTAGACATTACATCCGCC

GGACTGGACGGCAGCAATCCACAAGCCGCAGAAACGCTTGACCTCTTCGCCACCTATCTG

GGGCGGCTTGCGGGCGACCTTGCGCTCATTTTCATGGCGCATGGCGGCGTTTATCTTTCG

GGTGGCATCCCGGTGCGCATCCTTTCCGCCCTCAAGGCCGGTTCGTTCCGCGCAG

>'dnak-13'

TTGCTGAGTTCAAGAAGGAAAGTGGCATCGACTTGAAGAACGACAAGCTTGCCCTGCAGCGCCTCAAGGAAGCTGCCGAAAAGGCCAAGATCGAACTGTCGTCCTCGCAGCAGACCGAAATCAACCTGCCGTTCATCACGGCTGACCAGACTGGCCCGAAGCATCTGGCGATCAAGCTGTCGCGCGCCAAGTTTGAAAGCCTGGTCGATGATCTCGTGCAGCGCACGGTCGAGCCGTGCAAGGCGGCGCTCAAGGATGCCGGCCTCAAGGCTGGCGAAATTGACGAAGTGGTTCTGGTCGGCGGCATGACCCGCATGCCCAAGATTCAGGAAGTCGTGAAGGCCTTCTTCGGCAAGGAACCGCACAAGGGCGTGAACCCGGATGAAGTCGTGGCCATGGGCGCGGCGATCCAGGGCGGCGTTTTGCAGGGCGACGTCAAGGACGTGCTGCTGCTCGACGTGACCCCGCTT

>gyrB_1

GGGCACCCTCATCACGGCGCTTGGCACCTCCATCGGCAAGGATGAAACGCACGGCTTCAA

CGCCGACAAGCTGCGTTATCACAAGATCATCATCATGACCGACGCCGACGTCGATGGCGC

CCATATTCGTACGCTTCTGCTCACCTTCTTCTTCCGGCAGATGCCGGAACTGATCGAACG

CGGGCATATCTATATCGCGCAGCCGCCGCTCTATAAGGTGACACGCGGCAAGTCTTCGCA

ATATATCAAGAACGAAGCCGCCTTTGAGGATTTCCTCATCGAAACCGGCCTTGAAGAAAC

GACACTGGAACTGGTGACTGGCGAAATGCGCGCCGGGCCGGATTTGCGCTCGGTGGTGGA

GGATGCGCGCACGCTGCGTCAGCTTCTGCACGGCCTGCACACCCGCTATGACCGCAGCGT

GGTGGAACAGGCGGCAATTGCCGGCCTGCTCAACCCCGATGCCTCAAGG

>trpE_5

ATGCATTTCAATGGCGATATGAATACAGGGCTGACGCTGCGCACCATCCGCATCAAGGAT

GGTGTGGCGGAAATCCGTGCAGGGGCGACGCTTCTGTTCGATTCCAACCCTGACGAGGAA

GAAGCCGAGACCGAATTGAAGGCATCGGCCATGATTGCGGCTGTGCGGGACGCACAGAAG

AGCAATCAGATCGCGGAAGAAAGTGTGGCGGCAAAGGTGGGTGAGGGGGTTTCGATCCTG

CTGGTCGATCACGAGGATTCCTTCGTCCATACGCTTGCCAATTATTTCCGCCAGACGGGC

GCCAAGGTTTCCACCGTGCGTTCACCGGTGGCAGAGGAGATATTCGACCGCGTCAATCCC

GATCTGGTGGTGTTATCGCCGGGACCGGGCTCGCCGCAGGATTTCGATTGCAAGGCGACC

ATCGATAAGGCGCGCAAGCGCCAGCTTCCGATTTTTGGCGTCTGCCTCGGCCTTCAGGCC

CTGGCG

>cobQ_3

CGGCGGATCAGTGACCCGGCGGGTATTGAAGGCAATGTGCGCGATATCGAGGGGCTGGGC

CTTCTCGATATCGAGACGATGACGGAGCCGGAAAAAGTGGTTCGCAATGTTGAGGCGGTG

TCGCTGCTGCATGATGAGCCGCTGGAGGGCTATGAAATCCACATCGGGCGCACCAGCGGG

CCGGATATGGCGCGGCCATTTGCGCGTATCGGCGATCATGATGATGGGGCCGTCTCGCCC

GATGGTCGTATCATGGGAACCTATCTCCACGGTGTTTTCAGTGCGGATCGTTTCCGCCAC

CACTTTTTGCGCGCGCTGGGTGTGGAAGGCGGTCAGATGAATTATCGCGAGAGCGTCGAA

GAGGCTCTGGGCGAACTGGCTGAAGGGCTGGAAGCCTCGCTGGATATTGATGGCCTGTTT

GC

>omp25_8

TCTCGTAATCGTCTCGGCTGCGTTGCTGCCGTTCTCTGCGACCGCTTTTGCTGCCGACGC

CATCCAGGAACAGCCTCCGGTTCCGGCTCCGGTTGAAGTAGCTCCCCAGTATAGCTGGGC

TGGTGGCTATACCGGTCTTTACCTTGGCTACGGCTGGAACAAGGCCAAGACCAGCACCGT

TGGCAGCATCAAGCCTGACGATTGGAAGGCTGGCGCCTTTGCTGGCTGGAACTTCCAGCA

GGACCAGATCGTATACGGCGTTGAAGGTGATGCAGGTTATTCCTGGGCCAAGAAGTCCAA

GGACGGCCTGGAAGTCAAGCAGGGCTTTGAAGGCTCGCTGCGTGCCCGCGTTGGCTACGA

CCTGAACCCGGTTATGCCGTACCTCACGGCTGGTATTGCCGGTTCGCAGATCAAGCTTAA

CAACGGCTTGGACGACGAAAGCAAGTTCCGCGTGGGTTGGACGGCTGGTGCCGGTCTCGA

AGCCAAGCTG

>int_hyp_2

TAACGGTGGTATCATGCCGAGTAAAAGAGCGATCTTTACACCCTTGTCGATCCTGTTTCG

CCCCCGCCACAACACAGCCTGATCGGCAAGCTGTGCTTTGGTGGAGGCGCCGGGTACCGC

CCCCGGGTCCAATGGGTTTATTACACCGTCCGTTTATCACCATAGTCGGCTTGCGCCGAC

AGGACGTATATAGGCGTGGTTTTTACCGATTGGAAGGGGGCTTGTGCGTTTTCGCGCAAG

ACCGACAGAGGTGGTGCGGCCCTTCCGTTCATTTTCCATTGACAGCTTCCGCGCGCTGGT

CAATCCTCACAATATATCGGGATCGGCCTTGAAGAGGCTTGGCGCAGCCGGGGCGGAAAC

CATGGCTGAAACGGGGACGATATGCCCCATTCGAAGGAGAGTGGATATATGAGTGAATAT

CTCGCGGATG

ST76

>gap_1

CTCCGCTCCCGCAGACGGTGCCGATCTCACCGTCGTCTATGGTGTCAACAACGACAAGCT

GACGAAGGACCATCTGGTCATCTCCAACGCTTCGTGCACCACCAACTGCCTTGCGCCGGT

GGCTCAGGTTCTCAACGATACTATCGGTATCGAAAAGGGCTTCATGACCACGATCCACTC

CTATACGGGCGACCAGCCGACGCTGGACACCATGCACAAGGATCTCTACCGCGCCCGCGC

CGCTGCCCTTTCCATGATCCCGACCTCGACGGGTGCGGCCAAGGCCGTCGGTCTCGTTCT

GCCGGAACTGAAAGGCAAGCTCGACGGCGTTGCCATTCGCGTCCCGACCCCAAATGTCTC

GGTCGTTGATCTCACCTTCATCGCCAAGCGTGAAACCACCGTTGAAGAAGTCAACAATGC

GATCCGCGAAGCCGCCAATGGCCGCCTCAAGGGCATTCTCGGCTATACCGATGAGAAGCT

CGTCTCGCACGACTTCAACCACGATTCCCATTCCTCGGTCTTCCACACCGACCAGACCAA

GGTTATGGACGGCACCATGGTGCGTATCCTGTCGTGGTACGACAATGAA

>'aroA-8'

>'aroA-15'

TGATCCCTCGTCAACGGCTTTTCCGCTGGTGGCCGCCCTTCTGGTCGAAGGTTCGGACGTCACCATCCGCAATGTGCTGATGAACCCGACCCGCACCGGCCTGATCCTGACGTTGCAGGAAATGGGGGCGGATATCGAGATCATCGATCCACGCCTTGCCGGCGGCGAGGATGTCGCCGATCTGCGCGTCAGGGCCTCGAAGCTGAAAGGCGTTGTCGTTCCGCCGGAACGTGCGCCTTCGATGATCGATGAATATCCGGTTCTGGCCATTGCCGCGTCTTTTGCGGAAGGCGAAACCGTGATGGACGGTCTCGATGAACTGCGCGTCAAGGAATCGGATCGTCTGGCGGCCGTTGCGCGCGGCCTTGAAGCCAATGGTGTCGATTGTACCGAAGGCGAGATGTCGCTGACGGTTCGTGGCCGCCCCGGCGGCAAGGGGCTGGGCGGTGGCACGGTTGCAACCCACCTCGACCACCGCATCGCGATGAGTTTCCTCGTCATGGGCCTTGCATCGGAAAAGCCGGTTACGCTGGATGACAGCACCATGATCGCC

ACCTCTTTCCCG

>glk_4

AAACCGGAGGAGGCTGTTGCCACCCGCGTCGTGCTCGGCCCCGGCACGGGCCTTGGCGTG

GCAGGTCTGGTTCGCACACGTCATGCATGGGTTCCGGTTCCCGGTGAAGGCGGTCATATC

GATATCGGTCCACGCACCGAACGCGACTACCAGATTTTCCCGCATATCGAACGCATCGAA

GGGCGTGTCACCGGCGAGCAAATTCTTAGCGGGCGGGGCCTGCGCAACCTCTATCTGGGC

ATCTGCGCCGCCGACAAGATCACGCCCACCCTTGAGACGCCAGTAGACATTACATCCGCC

GGACTGGACGGCAGCAATCCACAAGCCGCAGAAACGCTTGACCTCTTCGCCACCTATCTG

GGGCGGCTTGCGGGCGACCTTGCGCTCATTTTCATGGCGCATGGCGGCGTTTATCTTTCG

GGTGGCATCCCGGTGCGCATCCTTTCCGCCCTCAAGGCCGGTTCGTTCCGCGCAG

>dnaK_1

TTGCCGAGTTCAAGAAGGAAAGTGGCATCGACCTGAAGAACGACAAGCTTGCCCTGCAGC

GCCTCAAGGAAGCTGCCGAAAAGGCCAAGATCGAACTGTCGTCCTCGCAGCAGACCGAAA

TCAACCTGCCGTTCATCACGGCTGACCAGACTGGCCCGAAGCATCTGGCGATCAAGCTGT

CGCGCGCCAAGTTTGAAAGCCTGGTCGATGATCTCGTGCAGCGCACGGTCGAGCCGTGCA

AGGCGGCGCTCAAGGATGCCGGCCTCAAGGCTGGCGAAATTGACGAAGTGGTTCTGGTTG

GCGGCATGACCCGCATGCCCAAGATTCAGGAAGTCGTGAAGGCCTTCTTCGGCAAGGAAC

CGCACAAGGGCGTGAACCCGGATGAAGTCGTGGCCATGGGCGCGGCGATCCAGGGCGGCG

TTTTGCAGGGCGACGTCAAGGACGTGCTGCTGCTCGACGTGACCCCGCTT

>gyrB_5

GGGCACCCTCATCACGGCGCTTGGCACCTCCATCGGCAAGGATGAAACGCACGGCTTCAA

CGCCGACAAACTGCGTTATCACAAGATCATCATCATGACCGACGCCGACGTCGATGGCGC

CCATATTCGTACGCTTCTGCTCACCTTCTTCTTCCGGCAGATGCCGGAACTGATCGAACG

CGGGCATATCTATATCGCGCAGCCGCCGCTCTATAAGGTGACACGCGGCAAGTCTTCGCA

ATATATCAAGAACGAAGCCGCCTTTGAAGATTTCCTCATCGAAACCGGCCTTGAAGAAAC

GACACTGGAACTGGTGACTGGCGAAATGCGCGCCGGGCCGGATTTGCGCTCGGTGGTGGA

GGATGCGCGCATGCTGCGTCAGCTTCTGCACGGCCTGCACACCCGCTATGACCGCAGCGT

GGTGGAACAGGCGGCAATTGCCGGCCTGCTCAACCCCGATGCCTCAAGG

>trpE_3

ATGCATTTCAATGGCGATATGAATACAGGGCTGACGCTGCGCACCATCCGCATCAAGGAT

GGTGTGGCGGAAATCCGTGCAGGGGCGACGCTTCTGTTCGATTCCAACCCTGACGAGGAA

GAAGCCGAGACCGAATTGAAGGCATCGGCCATGATTGCGGCTGTGCGGGACGCACAGAAG

AGCAATCAGATCGCGGAAGAAAGTGTGGCGGCAAAGGTGGGTGAGGGGGTTTCGATCCTG

CTGGTCGATCACGAGGATTCCTTCGTCCATACGCTTGCCAATTATTTCCGCCAGACGGGC

GCCAAGGTTTCCACCGTGCGTTCACCGGTGGCAGAGGAGATATTCGACCGCGTCAATCCC

GATCTGGTGGTGTTATCGCCGGGACCGGGCTCGCCGCAGGATTTCGATTGCAAGGCGACC

ATCGATAAGGCGCGCAAGCGCCAGCTTCCGATTTTTGGCGTCTGCCTCGGCCTTCAGGCA

CTGGCG

>cobQ_1

CGGCGGATCAGTGACCCGGCGGGTATTGAAGGCAATGTGCGCGATATCGAGGGGCTGGGC

CTTCTCGATATCGAGACGATGACGGAGCCGGAAAAAGTGGTTCGCAATGTTGAGGCGGTG

TCGCTGCTGCATGATGAGCCGCTGGAGGGCTATGAAATCCACATCGGGCGCACCAGCGGG

CCGGATATGGCGCGGCCATTTGCGCGTATCGGCGATCATGATGATGGGGCCGTCTCGCCC

GATGGTCGTATCATGGGAACCTATCTCCACGGTATTTTCAGTGCGGATCGTTTCCGCCAC

CACTTTTTGCGCGCGCTGGGTGTGGAAGGCGGCCAGATGAATTATCGCGAGAGCGTCGAA

GAGGCTCTGGGCGAACTGGCTGAAGGGCTGGAAGCCTCGCTGGATATTGATGGCCTGTTT

GC

>omp25_2

TCTCGTAATCGTCTCGGCTGCGCTGCTGCCGTTCTCTGCGACCGCTTTTGCTGCCGACGC

CATCCAGGAACAGCCTCCGGTTCCGGCTCCGGTTGAAGTAGCTCCCCAGTATAGCTGGGC

TGGTGGCTATACCGGTCTTTACCTTGGCTACGGCTGGAACAAGGCCAAGACCAGCACCGT

TGGCAGCATCAAGCCTGACGATTGGAAGGCTGGCGCCTTTGCTGGCTGGAACTTCCAGCA

GGACCAGATCGTATACGGTGTTGAAGGTGATGCAGGTTATTCCTGGGCCAAGAAGTCCAA

GGACGGCCTGGAAGTCAAGCAGGGCTTTGAAGGCTCGCTGCGTGCCCGCGTCGGCTACGA

CCTGAACCCGGTTATGCCGTACCTCACGGCTGGTATTGCCGGTTCGCAGATCAAGCTTAA

CAACGGCTTGGACGACGAAAGCAAGTTCCGCGTGGGTTGGACGGCTGGTGCCGGTCTCGA

AGCCAAGCTG

>int_hyp_4

TAACGGTGGTATCATACCGAGTAAAAGAGCGATCTTTACACCCTTGTCGATCCTGTTTCG

CCCCCGCCACAACACAGCCTGATCGGCAAGCTGTGCTGTGGTGGAGGCGCCGGGTACCGC

CCCCGGGTCCAATGGGTTTATTACACCGTCCGTTTATCACCATAGTCGGCTTGCGCCGAC

AGGACGTATATAGGCGTGGTTTTTACCGATTGGAAGGGGGCTTGTGCGTTTTCGCGCAAG

ACCGACAGAGGTGGTGCGGCCCTTCCGTTCATTTTCCATTGACAGCTTCCGCGTGCTGGT

CAATCCTCACAATATATCGGGATCGGCCTTGAAGAGGCTTGGCGCAGCCGGGGCGGAAAC

CATGGCTGAAACGGGGACGATATGCCCCAATCGAAGGAGAGTGGATATATGAGTGAATAT

CTCGCGGATG

ST77

>gap_1

CTCCGCTCCCGCAGACGGTGCCGATCTCACCGTCGTCTATGGTGTCAACAACGACAAGCT

GACGAAGGACCATCTGGTCATCTCCAACGCTTCGTGCACCACCAACTGCCTTGCGCCGGT

GGCTCAGGTTCTCAACGATACTATCGGTATCGAAAAGGGCTTCATGACCACGATCCACTC

CTATACGGGCGACCAGCCGACGCTGGACACCATGCACAAGGATCTCTACCGCGCCCGCGC

CGCTGCCCTTTCCATGATCCCGACCTCGACGGGTGCGGCCAAGGCCGTCGGTCTCGTTCT

GCCGGAACTGAAAGGCAAGCTCGACGGCGTTGCCATTCGCGTCCCGACCCCAAATGTCTC

GGTCGTTGATCTCACCTTCATCGCCAAGCGTGAAACCACCGTTGAAGAAGTCAACAATGC

GATCCGCGAAGCCGCCAATGGCCGCCTCAAGGGCATTCTCGGCTATACCGATGAGAAGCT

CGTCTCGCACGACTTCAACCACGATTCCCATTCCTCGGTCTTCCACACCGACCAGACCAA

GGTTATGGACGGCACCATGGTGCGTATCCTGTCGTGGTACGACAATGAA

>'aroA-15'

TGATCCCTCGTCAACGGCTTTTCCGCTGGTGGCCGCCCTTCTGGTCGAAGGTTCGGACGTCACCATCCGCAATGTGCTGATGAACCCGACCCGCACCGGCCTGATCCTGACGTTGCAGGAAATGGGGGCGGATATCGAGATCATCGATCCACGCCTTGCCGGCGGCGAGGATGTCGCCGATCTGCGCGTCAGGGCCTCGAAGCTGAAAGGCGTTGTCGTTCCGCCGGAACGTGCGCCTTCGATGATCGATGAATATCCGGTTCTGGCCATTGCCGCGTCTTTTGCGGAAGGCGAAACCGTGATGGACGGTCTCGATGAACTGCGCGTCAAGGAATCGGATCGTCTGGCGGCCGTTGCGCGCGGCCTTGAAGCCAATGGTGTCGATTGTACCGAAGGCGAGATGTCGCTGACGGTTCGTGGCCGCCCCGGCGGCAAGGGGCTGGGCGGTGGCACGGTTGCAACCCACCTCGACCACCGCATCGCGATGAGTTTCCTCGTCATGGGCCTTGCATCGGAAAAGCCGGTTACGCTGGATGACAGCACCATGATCGCCACCTCTTTCCCG

>glk_4

AAACCGGAGGAGGCTGTTGCCACCCGCGTCGTGCTCGGCCCCGGCACGGGCCTTGGCGTG

GCAGGTCTGGTTCGCACACGTCATGCATGGGTTCCGGTTCCCGGTGAAGGCGGTCATATC

GATATCGGTCCACGCACCGAACGCGACTACCAGATTTTCCCGCATATCGAACGCATCGAA

GGGCGTGTCACCGGCGAGCAAATTCTTAGCGGGCGGGGCCTGCGCAACCTCTATCTGGGC

ATCTGCGCCGCCGACAAGATCACGCCCACCCTTGAGACGCCAGTAGACATTACATCCGCC

GGACTGGACGGCAGCAATCCACAAGCCGCAGAAACGCTTGACCTCTTCGCCACCTATCTG

GGGCGGCTTGCGGGCGACCTTGCGCTCATTTTCATGGCGCATGGCGGCGTTTATCTTTCG

GGTGGCATCCCGGTGCGCATCCTTTCCGCCCTCAAGGCCGGTTCGTTCCGCGCAG

>dnaK_1

TTGCCGAGTTCAAGAAGGAAAGTGGCATCGACCTGAAGAACGACAAGCTTGCCCTGCAGC

GCCTCAAGGAAGCTGCCGAAAAGGCCAAGATCGAACTGTCGTCCTCGCAGCAGACCGAAA

TCAACCTGCCGTTCATCACGGCTGACCAGACTGGCCCGAAGCATCTGGCGATCAAGCTGT

CGCGCGCCAAGTTTGAAAGCCTGGTCGATGATCTCGTGCAGCGCACGGTCGAGCCGTGCA

AGGCGGCGCTCAAGGATGCCGGCCTCAAGGCTGGCGAAATTGACGAAGTGGTTCTGGTTG

GCGGCATGACCCGCATGCCCAAGATTCAGGAAGTCGTGAAGGCCTTCTTCGGCAAGGAAC

CGCACAAGGGCGTGAACCCGGATGAAGTCGTGGCCATGGGCGCGGCGATCCAGGGCGGCG

TTTTGCAGGGCGACGTCAAGGACGTGCTGCTGCTCGACGTGACCCCGCTT

>gyrB_4

GGGCACCCTCATCACGGCGCTTGGCACCTCCATCGGCAAGGATGAAACGCACGGCTTCAA

CGCCGACAAACTGCGTTATCACAAGATCATCATCATGACCGACGCCGACGTCGATGGCGC

CCATATTCGTACGCTTCTGCTCACCTTCTTCTTCCGGCAGATGCCGGAACTGATCGAACG

CGGGCATATCTATATCGCGCAGCCGCCGCTCTATAAGGTGACACGCGGCAAGTCTTCGCA

ATATATCAAGAACGAAGCCGCCTTTGAGGATTTCCTCATCGAAACCGGCCTTGAAGAAAC

GACACTGGAACTGGTGACTGGCGAAATGCGCGCCGGGCCGGATTTGCGCTCGGTGGTGGA

GGATGCGCGCATGCTGCGTCAGCTTCTGCACGGCCTGCACACCCGCTATGACCGCAGCGT

GGTGGAACAGGCGGCAATTGCCGGCCTGCTCAACCCCGATGCCTCAAGG

>trpE_3

ATGCATTTCAATGGCGATATGAATACAGGGCTGACGCTGCGCACCATCCGCATCAAGGAT

GGTGTGGCGGAAATCCGTGCAGGGGCGACGCTTCTGTTCGATTCCAACCCTGACGAGGAA

GAAGCCGAGACCGAATTGAAGGCATCGGCCATGATTGCGGCTGTGCGGGACGCACAGAAG

AGCAATCAGATCGCGGAAGAAAGTGTGGCGGCAAAGGTGGGTGAGGGGGTTTCGATCCTG

CTGGTCGATCACGAGGATTCCTTCGTCCATACGCTTGCCAATTATTTCCGCCAGACGGGC

GCCAAGGTTTCCACCGTGCGTTCACCGGTGGCAGAGGAGATATTCGACCGCGTCAATCCC

GATCTGGTGGTGTTATCGCCGGGACCGGGCTCGCCGCAGGATTTCGATTGCAAGGCGACC

ATCGATAAGGCGCGCAAGCGCCAGCTTCCGATTTTTGGCGTCTGCCTCGGCCTTCAGGCA

CTGGCG

>cobQ_1

CGGCGGATCAGTGACCCGGCGGGTATTGAAGGCAATGTGCGCGATATCGAGGGGCTGGGC

CTTCTCGATATCGAGACGATGACGGAGCCGGAAAAAGTGGTTCGCAATGTTGAGGCGGTG

TCGCTGCTGCATGATGAGCCGCTGGAGGGCTATGAAATCCACATCGGGCGCACCAGCGGG

CCGGATATGGCGCGGCCATTTGCGCGTATCGGCGATCATGATGATGGGGCCGTCTCGCCC

GATGGTCGTATCATGGGAACCTATCTCCACGGTATTTTCAGTGCGGATCGTTTCCGCCAC

CACTTTTTGCGCGCGCTGGGTGTGGAAGGCGGCCAGATGAATTATCGCGAGAGCGTCGAA

GAGGCTCTGGGCGAACTGGCTGAAGGGCTGGAAGCCTCGCTGGATATTGATGGCCTGTTT

GC

>omp25_2

TCTCGTAATCGTCTCGGCTGCGCTGCTGCCGTTCTCTGCGACCGCTTTTGCTGCCGACGC

CATCCAGGAACAGCCTCCGGTTCCGGCTCCGGTTGAAGTAGCTCCCCAGTATAGCTGGGC

TGGTGGCTATACCGGTCTTTACCTTGGCTACGGCTGGAACAAGGCCAAGACCAGCACCGT

TGGCAGCATCAAGCCTGACGATTGGAAGGCTGGCGCCTTTGCTGGCTGGAACTTCCAGCA

GGACCAGATCGTATACGGTGTTGAAGGTGATGCAGGTTATTCCTGGGCCAAGAAGTCCAA

GGACGGCCTGGAAGTCAAGCAGGGCTTTGAAGGCTCGCTGCGTGCCCGCGTCGGCTACGA

CCTGAACCCGGTTATGCCGTACCTCACGGCTGGTATTGCCGGTTCGCAGATCAAGCTTAA

CAACGGCTTGGACGACGAAAGCAAGTTCCGCGTGGGTTGGACGGCTGGTGCCGGTCTCGA

AGCCAAGCTG

>int_hyp_1

TAACGGTGGTATCATGCCGAGTAAAAGAGCGATCTTTACACCCTTGTCGATCCTGTTTCG

CCCCCGCCACAACACAGCCTGATCGGCAAGCTGTGCTGTGGTGGAGGCGCCGGGTACCGC

CCCCGGGTCCAATGGGTTTATTACACCGTCCGTTTATCACCATAGTCGGCTTGCGCCGAC

AGGACGTATATAGGCGTGGTTTTTACCGATTGGAAGGGGGCTTGTGCGTTTTCGCGCAAG

ACCGACAGAGGTGGTGCGGCCCTTCCGTTCATTTTCCATTGACAGCTTCCGCGTGCTGGT

CAATCCTCACAATATATCGGGATCGGCCTTGAAGAGGCTTGGCGCAGCCGGGGCGGAAAC

CATGGCTGAAACGGGGACGATATGCCCCAATCGAAGGAGAGTGGATATATGAGTGAATAT

CTCGCGGATG

ST78

>gap_1

CTCCGCTCCCGCAGACGGTGCCGATCTCACCGTCGTCTATGGTGTCAACAACGACAAGCT

GACGAAGGACCATCTGGTCATCTCCAACGCTTCGTGCACCACCAACTGCCTTGCGCCGGT

GGCTCAGGTTCTCAACGATACTATCGGTATCGAAAAGGGCTTCATGACCACGATCCACTC

CTATACGGGCGACCAGCCGACGCTGGACACCATGCACAAGGATCTCTACCGCGCCCGCGC

CGCTGCCCTTTCCATGATCCCGACCTCGACGGGTGCGGCCAAGGCCGTCGGTCTCGTTCT

GCCGGAACTGAAAGGCAAGCTCGACGGCGTTGCCATTCGCGTCCCGACCCCAAATGTCTC

GGTCGTTGATCTCACCTTCATCGCCAAGCGTGAAACCACCGTTGAAGAAGTCAACAATGC

GATCCGCGAAGCCGCCAATGGCCGCCTCAAGGGCATTCTCGGCTATACCGATGAGAAGCT

CGTCTCGCACGACTTCAACCACGATTCCCATTCCTCGGTCTTCCACACCGACCAGACCAA

GGTTATGGACGGCACCATGGTGCGTATCCTGTCGTGGTACGACAATGAA

>'aroA-15

TGATCCCTCGTCAACGGCTTTTCCGCTGGTGGCCGCCCTTCTGGTCGAAGGTTCGGACGTCACCATCCGCAATGTGCTGATGAACCCGACCCGCACCGGCCTGATCCTGACGTTGCAGGAAATGGGGGCGGATATCGAGATCATCGATCCACGCCTTGCCGGCGGCGAGGATGTCGCCGATCTGCGCGTCAGGGCCTCGAAGCTGAAAGGCGTTGTCGTTCCGCCGGAACGTGCGCCTTCGATGATCGATGAATATCCGGTTCTGGCCATTGCCGCGTCTTTTGCGGAAGGCGAAACCGTGATGGACGGTCTCGATGAACTGCGCGTCAAGGAATCGGATCGTCTGGCGGCCGTTGCGCGCGGCCTTGAAGCCAATGGTGTCGATTGTACCGAAGGCGAGATGTCGCTGACGGTTCGTGGCCGCCCCGGCGGCAAGGGGCTGGGCGGTGGCACGGTTGCAACCCACCTCGACCACCGCATCGCGATGAGTTTCCTCGTCATGGGCCTTGCATCGGAAAAGCCGGTTACGCTGGATGACAGCACCATGATCGCCACCTCTTTCCCG

>glk_4

AAACCGGAGGAGGCTGTTGCCACCCGCGTCGTGCTCGGCCCCGGCACGGGCCTTGGCGTG

GCAGGTCTGGTTCGCACACGTCATGCATGGGTTCCGGTTCCCGGTGAAGGCGGTCATATC

GATATCGGTCCACGCACCGAACGCGACTACCAGATTTTCCCGCATATCGAACGCATCGAA

GGGCGTGTCACCGGCGAGCAAATTCTTAGCGGGCGGGGCCTGCGCAACCTCTATCTGGGC

ATCTGCGCCGCCGACAAGATCACGCCCACCCTTGAGACGCCAGTAGACATTACATCCGCC

GGACTGGACGGCAGCAATCCACAAGCCGCAGAAACGCTTGACCTCTTCGCCACCTATCTG

GGGCGGCTTGCGGGCGACCTTGCGCTCATTTTCATGGCGCATGGCGGCGTTTATCTTTCG

GGTGGCATCCCGGTGCGCATCCTTTCCGCCCTCAAGGCCGGTTCGTTCCGCGCAG

>dnaK_1

TTGCCGAGTTCAAGAAGGAAAGTGGCATCGACCTGAAGAACGACAAGCTTGCCCTGCAGC

GCCTCAAGGAAGCTGCCGAAAAGGCCAAGATCGAACTGTCGTCCTCGCAGCAGACCGAAA

TCAACCTGCCGTTCATCACGGCTGACCAGACTGGCCCGAAGCATCTGGCGATCAAGCTGT

CGCGCGCCAAGTTTGAAAGCCTGGTCGATGATCTCGTGCAGCGCACGGTCGAGCCGTGCA

AGGCGGCGCTCAAGGATGCCGGCCTCAAGGCTGGCGAAATTGACGAAGTGGTTCTGGTTG

GCGGCATGACCCGCATGCCCAAGATTCAGGAAGTCGTGAAGGCCTTCTTCGGCAAGGAAC

CGCACAAGGGCGTGAACCCGGATGAAGTCGTGGCCATGGGCGCGGCGATCCAGGGCGGCG

TTTTGCAGGGCGACGTCAAGGACGTGCTGCTGCTCGACGTGACCCCGCTT

>gyrB_4

GGGCACCCTCATCACGGCGCTTGGCACCTCCATCGGCAAGGATGAAACGCACGGCTTCAA

CGCCGACAAACTGCGTTATCACAAGATCATCATCATGACCGACGCCGACGTCGATGGCGC

CCATATTCGTACGCTTCTGCTCACCTTCTTCTTCCGGCAGATGCCGGAACTGATCGAACG

CGGGCATATCTATATCGCGCAGCCGCCGCTCTATAAGGTGACACGCGGCAAGTCTTCGCA

ATATATCAAGAACGAAGCCGCCTTTGAGGATTTCCTCATCGAAACCGGCCTTGAAGAAAC

GACACTGGAACTGGTGACTGGCGAAATGCGCGCCGGGCCGGATTTGCGCTCGGTGGTGGA

GGATGCGCGCATGCTGCGTCAGCTTCTGCACGGCCTGCACACCCGCTATGACCGCAGCGT

GGTGGAACAGGCGGCAATTGCCGGCCTGCTCAACCCCGATGCCTCAAGG

>trpE_3

ATGCATTTCAATGGCGATATGAATACAGGGCTGACGCTGCGCACCATCCGCATCAAGGAT

GGTGTGGCGGAAATCCGTGCAGGGGCGACGCTTCTGTTCGATTCCAACCCTGACGAGGAA

GAAGCCGAGACCGAATTGAAGGCATCGGCCATGATTGCGGCTGTGCGGGACGCACAGAAG

AGCAATCAGATCGCGGAAGAAAGTGTGGCGGCAAAGGTGGGTGAGGGGGTTTCGATCCTG

CTGGTCGATCACGAGGATTCCTTCGTCCATACGCTTGCCAATTATTTCCGCCAGACGGGC

GCCAAGGTTTCCACCGTGCGTTCACCGGTGGCAGAGGAGATATTCGACCGCGTCAATCCC

GATCTGGTGGTGTTATCGCCGGGACCGGGCTCGCCGCAGGATTTCGATTGCAAGGCGACC

ATCGATAAGGCGCGCAAGCGCCAGCTTCCGATTTTTGGCGTCTGCCTCGGCCTTCAGGCA

CTGGCG

>cobQ_5

CGGCGGATCAGTGACCCGGCGGGTATTGAAGGCAATGTGCGCGATATCGAGGGGCTGGGC

CTTCTCGATATCGAGACGATGATGGAGCCGGAAAAAGTGGTTCGCAATGTTGAGGCGGTG

TCGCTGCTGCATGATGAGCCGCTGGAGGGCTATGAAATCCACATCGGGCGCACCAGCGGG

CCGGATATGGCGCGGCCATTTGCGCGTATCGGCGATCATGATGATGGGGCCGTCTCGCCC

GATGGTCGTATCATGGGAACCTATCTCCACGGTGTTTTCAGTGCGGATCGTTTCCGCCAC

CACTTTTTGCGCGCGCTGGGTGTGGAAGGCGGCCAGATGAATTATCGCGAGAGCGTCGAA

GAGGCTCTGGACGAACTGGCTGAAGGGCTGGAAGCCTCGCTGGATATTGATGGCCTGTTT

GC

>omp25_2

TCTCGTAATCGTCTCGGCTGCGCTGCTGCCGTTCTCTGCGACCGCTTTTGCTGCCGACGC

CATCCAGGAACAGCCTCCGGTTCCGGCTCCGGTTGAAGTAGCTCCCCAGTATAGCTGGGC

TGGTGGCTATACCGGTCTTTACCTTGGCTACGGCTGGAACAAGGCCAAGACCAGCACCGT

TGGCAGCATCAAGCCTGACGATTGGAAGGCTGGCGCCTTTGCTGGCTGGAACTTCCAGCA

GGACCAGATCGTATACGGTGTTGAAGGTGATGCAGGTTATTCCTGGGCCAAGAAGTCCAA

GGACGGCCTGGAAGTCAAGCAGGGCTTTGAAGGCTCGCTGCGTGCCCGCGTCGGCTACGA

CCTGAACCCGGTTATGCCGTACCTCACGGCTGGTATTGCCGGTTCGCAGATCAAGCTTAA

CAACGGCTTGGACGACGAAAGCAAGTTCCGCGTGGGTTGGACGGCTGGTGCCGGTCTCGA

AGCCAAGCTG

>int_hyp_1

TAACGGTGGTATCATGCCGAGTAAAAGAGCGATCTTTACACCCTTGTCGATCCTGTTTCG

CCCCCGCCACAACACAGCCTGATCGGCAAGCTGTGCTGTGGTGGAGGCGCCGGGTACCGC

CCCCGGGTCCAATGGGTTTATTACACCGTCCGTTTATCACCATAGTCGGCTTGCGCCGAC

AGGACGTATATAGGCGTGGTTTTTACCGATTGGAAGGGGGCTTGTGCGTTTTCGCGCAAG

ACCGACAGAGGTGGTGCGGCCCTTCCGTTCATTTTCCATTGACAGCTTCCGCGTGCTGGT

CAATCCTCACAATATATCGGGATCGGCCTTGAAGAGGCTTGGCGCAGCCGGGGCGGAAAC

CATGGCTGAAACGGGGACGATATGCCCCAATCGAAGGAGAGTGGATATATGAGTGAATAT

CTCGCGGATG

ST79

>gap_2

CTCCGCTCCCGCAGACGGTGCCGATCTCACCGTCGTCTATGGTGTCAACAACGACAAGCT

GACGAAGGACCATCTGGTCATCTCCAACGCTTCGTGTACCACCAACTGCCTTGCGCCGGT

GGCTCAGGTTCTCAACGATACTATCGGTATCGAAAAGGGCTTCATGACCACGATCCACTC

CTATACGGGCGACCAGCCGACGCTGGACACCATGCACAAGGATCTCTACCGCGCCCGCGC

CGCTGCCCTTTCCATGATCCCGACCTCGACGGGTGCGGCCAAGGCCGTCGGTCTCGTTCT

GCCGGAACTGAAAGGCAAGCTCGACGGCGTTGCCATTCGCGTCCCGACCCCAAATGTCTC

GGTCGTTGATCTCACCTTCATCGCCAAGCGTGAAACCACCGTTGAAGAAGTCAACAATGC

GATCCGCGAAGCCGCCAATGGCCGCCTCAAGGGCATTCTCGGCTATACCGATGAGAAGCT

CGTCTCGCACGACTTCAACCACGATTCCCATTCCTCGGTCTTCCACACCGACCAGACCAA

GGTTATGGACGGCACCATGGTGCGTATCCTGTCGTGGTACGACAATGAA

>aroA_1

TGATCCCTCGTCAACGGCTTTTCCGCTGGTGGCCGCCCTTCTGGTCGAAGGTTCGGAGGT

CACCATCCGCAATGTGCTGATGAACCCGACCCGCACCGGCCTGATCCTGACGTTGCAGGA

AATGGGGGCGGATATCGAGATCATCGATCCACGCCTTGCCGGCGGCGAGGATGTCGCCGA

TCTGCGCGTCAAGGCCTCGAAGCTGAAAGGCGTTGTCGTTCCGCCGGAACGTGCGCCTTC

GATGATCGATGAATATCCGGTTCTGGCCATTGCCGCGTCTTTTGCGGAAGGCGAAACCGT

GATGGACGGTCTCGATGAACTGCGCGTCAAGGAATCGGATCGTCTGGCGGCCGTTGCGCG

CGGCCTTGAAGCCAATGGTGTCGATTGTACCGAAGGCGAGATGTCGCTGACGGTTCGTGG

CCGCCCCGGCGGCAAGGGGCTGGGCGGTGGCACGGTTGCAACCCACCTCGACCACCGCAT

CGCGATGAGTTTCCTCGTCATGGGCCTTGCATCGGAAAAGCCGGTTACGGTGGATGACAG

CACCATGATCGCCACCTCTTTCCCG

>glk_1

AAACCGGAGGAGGCTGTTGCCACCCGCGTCGTGCTCGGCCCCGGCACGGGCCTTGGCGTG

GCAGGTCTGTTTCGCACACGTCATGCATGGGTTCCGGTTCCCGGTGAAGGCGGTCATATC

GATATCGGTCCACGCACCGAACGCGACTACCAGATTTTCCCGCATATCGAACGCATCGAA

GGGCGTGTCACCGGCGAGCAAATTCTTAGCGGGCGGGGCCTGCGCAACCTCTATCTGGGC

ATCTGCGCCGCCGACAAGATCACGCCCACCCTTGAGACGCCAGTAGACATTACATCCGCC

GGACTGGACGGCAGCAATCCACAAGCCGCAGAAACGCTTGACCTCTTCGCCACCTATCTG

GGGCGGCTTGCGGGCGACCTTGCGCTCATTTTCATGGCGCATGGCGGCGTTTATCTTTCG

GGTGGCATCCCGGTGCGCATCCTTTCCGCCCTCAAGGCCGGTTCGTTCCGCGCAA

>dnaK_2

TTGCCGAGTTCAAGAAGGAAAGTGGCATCGACCTGAAGAACGACAAGCTTGCCCTGCAGC

GCCTCAAGGAAGCTGCCGAAAAGGCCAAGATCGAACTGTCGTCCTCGCAGCAGACCGAAA

TCAACCTGCCGTTCATCACGGCTGACCAGACTGGCCCGAAGCATCTGGCGATCAAGCTGT

CGCGCGCCAAGTTTGAAAGCCTGGTCGATGATCTCGTGCAGCGCACGGTCGAGCCGTGCA

AGGCGGCGCTCAAGGATGCCGGCCTCAAGGCTGGCGAAATTGACGAAGTGGTTCTGGTCG

GCGGCATGACCCGCATGCCCAAGATTCAGGAAGTCGTGAAGGCCTTCTTCGGCAAGGAAC

CGCACAAGGGCGTGAACCCGGATGAAGTCGTGGCCATGGGCGCGGCGATCCAGGGCGGCG

TTTTGCAGGGCGACGTCAAGGACGTGCTGCTGCTCGACGTGACCCCGCTT

>gyrB_1

GGGCACCCTCATCACGGCGCTTGGCACCTCCATCGGCAAGGATGAAACGCACGGCTTCAA

CGCCGACAAGCTGCGTTATCACAAGATCATCATCATGACCGACGCCGACGTCGATGGCGC

CCATATTCGTACGCTTCTGCTCACCTTCTTCTTCCGGCAGATGCCGGAACTGATCGAACG

CGGGCATATCTATATCGCGCAGCCGCCGCTCTATAAGGTGACACGCGGCAAGTCTTCGCA

ATATATCAAGAACGAAGCCGCCTTTGAGGATTTCCTCATCGAAACCGGCCTTGAAGAAAC

GACACTGGAACTGGTGACTGGCGAAATGCGCGCCGGGCCGGATTTGCGCTCGGTGGTGGA

GGATGCGCGCACGCTGCGTCAGCTTCTGCACGGCCTGCACACCCGCTATGACCGCAGCGT

GGTGGAACAGGCGGCAATTGCCGGCCTGCTCAACCCCGATGCCTCAAGG

>trpE_4

ATGCATTTCAATGGCGATATGAATACAGGGCTGACGCTGCGCACCATCCGCATCAAGGAT

GGTGTGGCGGAAATCCGTGCAGGGGCGACGCTTCTGTTCGATTCCAACCCTGACGAGGAA

GAAGACGAGACCGAATTGAAGGCATCGGCCATGATTGCGGCTGTGCGGGACGCACAGAAG

AGCAATCAGATCGCGGAAGAAAGTGTGGCGGCAAAGGTGGGTGAGGGGGTTTCGATCCTG

CTGGTCGATCACGAGGATTCCTTCGTCCATACGCTTGCCAATTATTTCCGCCAGACGGGC

GCCAAGGTTTCCACCGTGCGTTCACCGGTGGCAGAGGAGATATTCGACCGCGTCAATCCC

GATCTGGTGGTGTTATCGCCGGGACCGGGCTCGCCGCAGGATTTCGATTGCAAGGCGACC

ATCGATAAGGCGCGCAAGCGCCAGCTTCCGATTTTTGGCGTCTGCCTCGGCCTTCAGGCA

CTGGCG

>cobQ_3

CGGCGGATCAGTGACCCGGCGGGTATTGAAGGCAATGTGCGCGATATCGAGGGGCTGGGC

CTTCTCGATATCGAGACGATGACGGAGCCGGAAAAAGTGGTTCGCAATGTTGAGGCGGTG

TCGCTGCTGCATGATGAGCCGCTGGAGGGCTATGAAATCCACATCGGGCGCACCAGCGGG

CCGGATATGGCGCGGCCATTTGCGCGTATCGGCGATCATGATGATGGGGCCGTCTCGCCC

GATGGTCGTATCATGGGAACCTATCTCCACGGTGTTTTCAGTGCGGATCGTTTCCGCCAC

CACTTTTTGCGCGCGCTGGGTGTGGAAGGCGGTCAGATGAATTATCGCGAGAGCGTCGAA

GAGGCTCTGGGCGAACTGGCTGAAGGGCTGGAAGCCTCGCTGGATATTGATGGCCTGTTT

GC

>omp25_1

TCTCGTAATCGTCTCGGCTGCGCTGCTGCCGTTCTCTGCGACCGCTTTTGCTGCCGACGC

CATCCAGGAACAGCCTCCGGTTCCGGCTCCGGTTGAAGTAGCTCCCCAGTATAGCTGGGC

TGGTGGCTATACCGGTCTTTACCTTGGCTATGGCTGGAACAAGGCCAAGACCAGCACCGT

TGGCAGCATCAAGCCTGACGATTGGAAGGCTGGCGCCTTTGCTGGCTGGAACTTCCAGCA

GGACCAGATCGTATACGGTGTTGAAGGTGATGCAGGTTATTCCTGGGCCAAGAAGTCCAA

GGACGGCCTGGAAGTCAAGCAGGGCTTTGAAGGCTCGCTGCGTGCCCGCGTCGGCTACGA

CCTGAACCCGGTTATGCCGTACCTCACGGCTGGTATTGCCGGTTCGCAGATCAAGCTTAA

CAACGGCTTGGACGACGAAAGCAAGTTCCGCGTGGGTTGGACGGCTGGTGCCGGTCTCGA

AGCCAAGCTG

>int_hyp_1

TAACGGTGGTATCATGCCGAGTAAAAGAGCGATCTTTACACCCTTGTCGATCCTGTTTCG

CCCCCGCCACAACACAGCCTGATCGGCAAGCTGTGCTGTGGTGGAGGCGCCGGGTACCGC

CCCCGGGTCCAATGGGTTTATTACACCGTCCGTTTATCACCATAGTCGGCTTGCGCCGAC

AGGACGTATATAGGCGTGGTTTTTACCGATTGGAAGGGGGCTTGTGCGTTTTCGCGCAAG

ACCGACAGAGGTGGTGCGGCCCTTCCGTTCATTTTCCATTGACAGCTTCCGCGTGCTGGT

CAATCCTCACAATATATCGGGATCGGCCTTGAAGAGGCTTGGCGCAGCCGGGGCGGAAAC

CATGGCTGAAACGGGGACGATATGCCCCAATCGAAGGAGAGTGGATATATGAGTGAATAT

CTCGCGGATG

ST80

>gap_1

CTCCGCTCCCGCAGACGGTGCCGATCTCACCGTCGTCTATGGTGTCAACAACGACAAGCT

GACGAAGGACCATCTGGTCATCTCCAACGCTTCGTGCACCACCAACTGCCTTGCGCCGGT

GGCTCAGGTTCTCAACGATACTATCGGTATCGAAAAGGGCTTCATGACCACGATCCACTC

CTATACGGGCGACCAGCCGACGCTGGACACCATGCACAAGGATCTCTACCGCGCCCGCGC

CGCTGCCCTTTCCATGATCCCGACCTCGACGGGTGCGGCCAAGGCCGTCGGTCTCGTTCT

GCCGGAACTGAAAGGCAAGCTCGACGGCGTTGCCATTCGCGTCCCGACCCCAAATGTCTC

GGTCGTTGATCTCACCTTCATCGCCAAGCGTGAAACCACCGTTGAAGAAGTCAACAATGC

GATCCGCGAAGCCGCCAATGGCCGCCTCAAGGGCATTCTCGGCTATACCGATGAGAAGCT

CGTCTCGCACGACTTCAACCACGATTCCCATTCCTCGGTCTTCCACACCGACCAGACCAA

GGTTATGGACGGCACCATGGTGCGTATCCTGTCGTGGTACGACAATGAA

>'aroA-15'

TGATCCCTCGTCAACGGCTTTTCCGCTGGTGGCCGCCCTTCTGGTCGAAGGTTCGGACGTCACCATCCGCAATGTGCTGATGAACCCGACCCGCACCGGCCTGATCCTGACGTTGCAGGAAATGGGGGCGGATATCGAGATCATCGATCCACGCCTTGCCGGCGGCGAGGATGTCGCCGATCTGCGCGTCAGGGCCTCGAAGCTGAAAGGCGTTGTCGTTCCGCCGGAACGTGCGCCTTCGATGATCGATGAATATCCGGTTCTGGCCATTGCCGCGTCTTTTGCGGAAGGCGAAACCGTGATGGACGGTCTCGATGAACTGCGCGTCAAGGAATCGGATCGTCTGGCGGCCGTTGCGCGCGGCCTTGAAGCCAATGGTGTCGATTGTACCGAAGGCGAGATGTCGCTGACGGTTCGTGGCCGCCCCGGCGGCAAGGGGCTGGGCGGTGGCACGGTTGCAACCCACCTCGACCACCGCATCGCGATGAGTTTCCTCGTCATGGGCCTTGCATCGGAAAAGCCGGTTACGCTGGATGACAGCACCATGATCGCCACCTCTTTCCCG

>glk_4

AAACCGGAGGAGGCTGTTGCCACCCGCGTCGTGCTCGGCCCCGGCACGGGCCTTGGCGTG

GCAGGTCTGGTTCGCACACGTCATGCATGGGTTCCGGTTCCCGGTGAAGGCGGTCATATC

GATATCGGTCCACGCACCGAACGCGACTACCAGATTTTCCCGCATATCGAACGCATCGAA

GGGCGTGTCACCGGCGAGCAAATTCTTAGCGGGCGGGGCCTGCGCAACCTCTATCTGGGC

ATCTGCGCCGCCGACAAGATCACGCCCACCCTTGAGACGCCAGTAGACATTACATCCGCC

GGACTGGACGGCAGCAATCCACAAGCCGCAGAAACGCTTGACCTCTTCGCCACCTATCTG

GGGCGGCTTGCGGGCGACCTTGCGCTCATTTTCATGGCGCATGGCGGCGTTTATCTTTCG

GGTGGCATCCCGGTGCGCATCCTTTCCGCCCTCAAGGCCGGTTCGTTCCGCGCAG

>dnaK_1

TTGCCGAGTTCAAGAAGGAAAGTGGCATCGACCTGAAGAACGACAAGCTTGCCCTGCAGC

GCCTCAAGGAAGCTGCCGAAAAGGCCAAGATCGAACTGTCGTCCTCGCAGCAGACCGAAA

TCAACCTGCCGTTCATCACGGCTGACCAGACTGGCCCGAAGCATCTGGCGATCAAGCTGT

CGCGCGCCAAGTTTGAAAGCCTGGTCGATGATCTCGTGCAGCGCACGGTCGAGCCGTGCA

AGGCGGCGCTCAAGGATGCCGGCCTCAAGGCTGGCGAAATTGACGAAGTGGTTCTGGTTG

GCGGCATGACCCGCATGCCCAAGATTCAGGAAGTCGTGAAGGCCTTCTTCGGCAAGGAAC

CGCACAAGGGCGTGAACCCGGATGAAGTCGTGGCCATGGGCGCGGCGATCCAGGGCGGCG

TTTTGCAGGGCGACGTCAAGGACGTGCTGCTGCTCGACGTGACCCCGCTT

>gyrB_5

GGGCACCCTCATCACGGCGCTTGGCACCTCCATCGGCAAGGATGAAACGCACGGCTTCAA

CGCCGACAAACTGCGTTATCACAAGATCATCATCATGACCGACGCCGACGTCGATGGCGC

CCATATTCGTACGCTTCTGCTCACCTTCTTCTTCCGGCAGATGCCGGAACTGATCGAACG

CGGGCATATCTATATCGCGCAGCCGCCGCTCTATAAGGTGACACGCGGCAAGTCTTCGCA

ATATATCAAGAACGAAGCCGCCTTTGAAGATTTCCTCATCGAAACCGGCCTTGAAGAAAC

GACACTGGAACTGGTGACTGGCGAAATGCGCGCCGGGCCGGATTTGCGCTCGGTGGTGGA

GGATGCGCGCATGCTGCGTCAGCTTCTGCACGGCCTGCACACCCGCTATGACCGCAGCGT

GGTGGAACAGGCGGCAATTGCCGGCCTGCTCAACCCCGATGCCTCAAGG

>trpE_3

ATGCATTTCAATGGCGATATGAATACAGGGCTGACGCTGCGCACCATCCGCATCAAGGAT

GGTGTGGCGGAAATCCGTGCAGGGGCGACGCTTCTGTTCGATTCCAACCCTGACGAGGAA

GAAGCCGAGACCGAATTGAAGGCATCGGCCATGATTGCGGCTGTGCGGGACGCACAGAAG

AGCAATCAGATCGCGGAAGAAAGTGTGGCGGCAAAGGTGGGTGAGGGGGTTTCGATCCTG

CTGGTCGATCACGAGGATTCCTTCGTCCATACGCTTGCCAATTATTTCCGCCAGACGGGC

GCCAAGGTTTCCACCGTGCGTTCACCGGTGGCAGAGGAGATATTCGACCGCGTCAATCCC

GATCTGGTGGTGTTATCGCCGGGACCGGGCTCGCCGCAGGATTTCGATTGCAAGGCGACC

ATCGATAAGGCGCGCAAGCGCCAGCTTCCGATTTTTGGCGTCTGCCTCGGCCTTCAGGCA

CTGGCG

>cobQ_1

CGGCGGATCAGTGACCCGGCGGGTATTGAAGGCAATGTGCGCGATATCGAGGGGCTGGGC

CTTCTCGATATCGAGACGATGACGGAGCCGGAAAAAGTGGTTCGCAATGTTGAGGCGGTG

TCGCTGCTGCATGATGAGCCGCTGGAGGGCTATGAAATCCACATCGGGCGCACCAGCGGG

CCGGATATGGCGCGGCCATTTGCGCGTATCGGCGATCATGATGATGGGGCCGTCTCGCCC

GATGGTCGTATCATGGGAACCTATCTCCACGGTATTTTCAGTGCGGATCGTTTCCGCCAC

CACTTTTTGCGCGCGCTGGGTGTGGAAGGCGGCCAGATGAATTATCGCGAGAGCGTCGAA

GAGGCTCTGGGCGAACTGGCTGAAGGGCTGGAAGCCTCGCTGGATATTGATGGCCTGTTT

GC

>omp25_5

TCTCGTAATCGTCTCGGCTGCGCTGCTGCCGTTCTCTGCGACCGCTTTTGCTGCCGACGC

CATCCAGGAACAGCCTCCGGTTCCGGCTCCGGTTGAAGTAGCTCCCCAGTATAGCTGGGC

TGGTGGCTATACCGGTCTTTACCTTGGCTACGGCTGGAACAAGGCCAAGACCAGCACCGT

TGGCAGCATCAAGCCTGACGATTGGAAGGCTGGCGCCTTTGCTGGCTGGAACTTCCAGAA

GGACCAGATCGTATACGGTGTTGAAGGTGATGCAGGTTATTCCTGGGCCAAGAAGTCCAA

GGACGGCCCGGAAGTCAAGCAGGGCTTTGAAGGCTCGCTGCGTGCCCGCGTCGGCTACGA

CCTGAACCCGGTTATGCCGTACCTCACGGCTGGTATTGCCGGTTCGCAGATCAAGCTTAA

CAACGGCTTGGACGACGAAAGCAAGTTCCGCGTGGGTTGGACGGCTGGTGCCGGTCTCGA

AGCCAAGCTG

>int_hyp_4

TAACGGTGGTATCATACCGAGTAAAAGAGCGATCTTTACACCCTTGTCGATCCTGTTTCG

CCCCCGCCACAACACAGCCTGATCGGCAAGCTGTGCTGTGGTGGAGGCGCCGGGTACCGC

CCCCGGGTCCAATGGGTTTATTACACCGTCCGTTTATCACCATAGTCGGCTTGCGCCGAC

AGGACGTATATAGGCGTGGTTTTTACCGATTGGAAGGGGGCTTGTGCGTTTTCGCGCAAG

ACCGACAGAGGTGGTGCGGCCCTTCCGTTCATTTTCCATTGACAGCTTCCGCGTGCTGGT

CAATCCTCACAATATATCGGGATCGGCCTTGAAGAGGCTTGGCGCAGCCGGGGCGGAAAC

CATGGCTGAAACGGGGACGATATGCCCCAATCGAAGGAGAGTGGATATATGAGTGAATAT

CTCGCGGATG

ST81

>gap_3

CTCCGCTCCCGCAGACGGTGCCGATCTCACCGTCGTCTATGGTGTCAACAACGACAAGCT

GACGAAGGACCATCTGGTCATCTCCAACGCTTCGTGTACCACCAACTGCCTTGCGCCGGT

GGCTCAGGTTCTCAACGATACTATCGGTATCGAAAAGGGCTTTATGACCACGATCCACTC

CTATACGGGCGACCAGCCGACGCTGGACACCATGCACAAGGATCTCTACCGCGCCCGCGC

CGCTGCCCTTTCCATGATCCCGACCTCGACGGGTGCGGCCAAGGCCGTCGGTCTCGTTCT

GCCGGAACTGAAAGGCAAGCTCTACGGCGTTGCCATTCGCGTCCCGACCCCAAATGTCTC

GGTCGTTGATCTCACCTTCATCGCCAAGCGTGAAACCACCGTTGAAGAAGTCAACAATGC

GATCCGCGAAGCCGCCAATGGCCGCCTCAAGGGCATTCTCGGCTATACCGATGAGAAGCT

CGTCTCGCACGACTTCAACCACGATTCCCATTCCTCGGTCTTCCACACCGACCAGACCAA

GGTTATGGACGGCACCATGGTGCGTATCCTGTCGTGGTACGACAATGAA

>'aroA-17'

TGATCCCTCGTCAACGGCTTTTCCGCTGGTGGCCGCCCTTCTGGTTGAAGGTTCGGACGTCACCATCCGCAATGTGTTGATGAACCCGACCCGCACCGGCCTGATCCTGACGTTGCAGGAAATGGGGGCGGATATCGAGATCATCGATCCACGCCTTGCCGGCGGCGAGGATGTCGCCGATCTGCGCGTCAAGGCCTCGAAGCTGAAAGGCGTTGTCGTTCCGCCGGAACGTGCGCCTTCGATGATCGATGAATATCCGGTTCTGGCCATTGCCGCGTCTTTTGCGGAAGGCGAAACCGTGATGGACGGTCTCGATGAACTGCGCGTCAAGGAATCGGATCGTCTGGCGGCCGTTGCGCGCGGCCTTGAAGCCAATGGTGTCGATTGTACCGAAGGCGAGATGTCGCTGACGGTTCGTGGCCGCCCCGGCGGCAAGGGGCTGGGCGGTGGCACGGTTGGAACCCACCTCGACCACCGCATCGCGATGAGTTTCCTCGTCATGGGCCTTGCATCGGAAAAGCCGGTTACGGTGGATGACAGCACCATGATCGCC

ACCTCTTTCCCG

>glk_3

AAACCGGAGGAGGCTGTTGCCACCCGCGTCGTGCTCGGCCCCGGCACGGGCCTTGGCGTG

GCAGGTCTGGTTTGCACACGTCATGCATGGGTTCCGGTTCCCGGTGAAGGCGGTCATATC

GATATCGGTCCACGCACCGAACGCGACTACCAGATTTTCCCGCATATCGAACGCATCGAA

GGGCGTGTCACCGGCGAGCAAATTCTTAGCGGGCGGGGCCTGCGCAACCTCTATCTGGGC

ATCTGCGCGGCCGACAAGATCACGCCCACCCTTGAGACGCCAGTAGACATTACATCCGCC

GGACTGGACGGCAGCAATCCACAAGCCGCAGAAACGCTTGACCTCTTCGCCACCTATCTG

GGGCGGCTTGCGGGCGACCTTGCGCTCATTTTCATGGCGCATGGCGGCGTTTATCTTTCG

GGTGGCATCCCGGTGCGCATCCTTTCCGCCCTCAAGGCCGGTTCGTTCCGCGCAG

>dnaK_2

TTGCCGAGTTCAAGAAGGAAAGTGGCATCGACCTGAAGAACGACAAGCTTGCCCTGCAGC

GCCTCAAGGAAGCTGCCGAAAAGGCCAAGATCGAACTGTCGTCCTCGCAGCAGACCGAAA

TCAACCTGCCGTTCATCACGGCTGACCAGACTGGCCCGAAGCATCTGGCGATCAAGCTGT

CGCGCGCCAAGTTTGAAAGCCTGGTCGATGATCTCGTGCAGCGCACGGTCGAGCCGTGCA

AGGCGGCGCTCAAGGATGCCGGCCTCAAGGCTGGCGAAATTGACGAAGTGGTTCTGGTCG

GCGGCATGACCCGCATGCCCAAGATTCAGGAAGTCGTGAAGGCCTTCTTCGGCAAGGAAC

CGCACAAGGGCGTGAACCCGGATGAAGTCGTGGCCATGGGCGCGGCGATCCAGGGCGGCG

TTTTGCAGGGCGACGTCAAGGACGTGCTGCTGCTCGACGTGACCCCGCTT

>gyrB_1

GGGCACCCTCATCACGGCGCTTGGCACCTCCATCGGCAAGGATGAAACGCACGGCTTCAA

CGCCGACAAGCTGCGTTATCACAAGATCATCATCATGACCGACGCCGACGTCGATGGCGC

CCATATTCGTACGCTTCTGCTCACCTTCTTCTTCCGGCAGATGCCGGAACTGATCGAACG

CGGGCATATCTATATCGCGCAGCCGCCGCTCTATAAGGTGACACGCGGCAAGTCTTCGCA

ATATATCAAGAACGAAGCCGCCTTTGAGGATTTCCTCATCGAAACCGGCCTTGAAGAAAC

GACACTGGAACTGGTGACTGGCGAAATGCGCGCCGGGCCGGATTTGCGCTCGGTGGTGGA

GGATGCGCGCACGCTGCGTCAGCTTCTGCACGGCCTGCACACCCGCTATGACCGCAGCGT

GGTGGAACAGGCGGCAATTGCCGGCCTGCTCAACCCCGATGCCTCAAGG

>trpE_5

ATGCATTTCAATGGCGATATGAATACAGGGCTGACGCTGCGCACCATCCGCATCAAGGAT

GGTGTGGCGGAAATCCGTGCAGGGGCGACGCTTCTGTTCGATTCCAACCCTGACGAGGAA

GAAGCCGAGACCGAATTGAAGGCATCGGCCATGATTGCGGCTGTGCGGGACGCACAGAAG

AGCAATCAGATCGCGGAAGAAAGTGTGGCGGCAAAGGTGGGTGAGGGGGTTTCGATCCTG

CTGGTCGATCACGAGGATTCCTTCGTCCATACGCTTGCCAATTATTTCCGCCAGACGGGC

GCCAAGGTTTCCACCGTGCGTTCACCGGTGGCAGAGGAGATATTCGACCGCGTCAATCCC

GATCTGGTGGTGTTATCGCCGGGACCGGGCTCGCCGCAGGATTTCGATTGCAAGGCGACC

ATCGATAAGGCGCGCAAGCGCCAGCTTCCGATTTTTGGCGTCTGCCTCGGCCTTCAGGCC

CTGGCG

>cobQ_2

CGGCGGATCAGTGACCCTGCGGGTATTGAAGGCAATGTGCGCGATATCGAGGGGCTGGGC

CTTCTCGATATCGAGACGATGACGGAGCCGGAAAAAGTGGTTCGCAATGTTGAGGCGGTG

TCGCTGCTGCATGATGAGCCGCTGGAGGGCTATGAAATCCACATCGGGCGCACCAGCGGG

CCGGATATGGCGCGGCCATTTGCGCGTATCGGCGATCATGATGATGGGGCCGTCTCGCCC

GATGGTCGTATCATGGGAACCTATCTCCACGGTGTTTTCAGTGCGGATCGTTTCCGCCAC

CACTTTTTGCGCGCGCTGGGTGTGGAAGGCGGTCAGATGAATTATCGCGAGAGCGTCGAA

GAGGCTCTGGGCGAACTGGCTGAAGGGCTGGAAGCCTCGCTGGATATTGATGGCCTGTTT

GC

>omp25_10

TCTCGTAATCGTCTCGGCTGCGTTGCTGCCGTTCTCTGCGACCGCTTTTGCTGCCGACGC

CATCCAGGAACAGCCTCCGGTTCCGGCTCCGGTTGAAGTAGCTCCCCAGTATAGCTGGGC

TGGTGGCTATACCGGTCTTTACCTTGGCTACGGCTGGAACAAGGCCAAGACCAGCACCGT

TGGCAGCATCAAGCCTGACGATTGGAAGGCTGGCGCCTTTGCTGGCTGGAACTTCCAGCA

GGACCAGATCGTATACGGTGTTGAAGGTGATGCAGGTTATTCCTGGGCCAAGAAGTCCAA

GGACGGCCTGGAAGTCAAGCAGGGCTTTGAAGGCTCGCTGCGTGCCCGCGTTGGCTACGA

CCTGAACCCGGTTATGCCGTACCTCACGGCTGGTATTGCCGGTTCGCAGATCAAGCTTAA

CAACGGCTTGGACGACGAAAGCAAGTTCCGCGTGGGTTGGACGGCTGGTGCCGGTCTCGA

AGCCAAGCTG

>int_hyp_2

TAACGGTGGTATCATGCCGAGTAAAAGAGCGATCTTTACACCCTTGTCGATCCTGTTTCG

CCCCCGCCACAACACAGCCTGATCGGCAAGCTGTGCTTTGGTGGAGGCGCCGGGTACCGC

CCCCGGGTCCAATGGGTTTATTACACCGTCCGTTTATCACCATAGTCGGCTTGCGCCGAC

AGGACGTATATAGGCGTGGTTTTTACCGATTGGAAGGGGGCTTGTGCGTTTTCGCGCAAG

ACCGACAGAGGTGGTGCGGCCCTTCCGTTCATTTTCCATTGACAGCTTCCGCGCGCTGGT

CAATCCTCACAATATATCGGGATCGGCCTTGAAGAGGCTTGGCGCAGCCGGGGCGGAAAC

CATGGCTGAAACGGGGACGATATGCCCCATTCGAAGGAGAGTGGATATATGAGTGAATAT

CTCGCGGATG

ST82

>gap_2

CTCCGCTCCCGCAGACGGTGCCGATCTCACCGTCGTCTATGGTGTCAACAACGACAAGCT

GACGAAGGACCATCTGGTCATCTCCAACGCTTCGTGTACCACCAACTGCCTTGCGCCGGT

GGCTCAGGTTCTCAACGATACTATCGGTATCGAAAAGGGCTTCATGACCACGATCCACTC

CTATACGGGCGACCAGCCGACGCTGGACACCATGCACAAGGATCTCTACCGCGCCCGCGC

CGCTGCCCTTTCCATGATCCCGACCTCGACGGGTGCGGCCAAGGCCGTCGGTCTCGTTCT

GCCGGAACTGAAAGGCAAGCTCGACGGCGTTGCCATTCGCGTCCCGACCCCAAATGTCTC

GGTCGTTGATCTCACCTTCATCGCCAAGCGTGAAACCACCGTTGAAGAAGTCAACAATGC

GATCCGCGAAGCCGCCAATGGCCGCCTCAAGGGCATTCTCGGCTATACCGATGAGAAGCT

CGTCTCGCACGACTTCAACCACGATTCCCATTCCTCGGTCTTCCACACCGACCAGACCAA

GGTTATGGACGGCACCATGGTGCGTATCCTGTCGTGGTACGACAATGAA

>aroA_1

TGATCCCTCGTCAACGGCTTTTCCGCTGGTGGCCGCCCTTCTGGTCGAAGGTTCGGAGGT

CACCATCCGCAATGTGCTGATGAACCCGACCCGCACCGGCCTGATCCTGACGTTGCAGGA

AATGGGGGCGGATATCGAGATCATCGATCCACGCCTTGCCGGCGGCGAGGATGTCGCCGA

TCTGCGCGTCAAGGCCTCGAAGCTGAAAGGCGTTGTCGTTCCGCCGGAACGTGCGCCTTC

GATGATCGATGAATATCCGGTTCTGGCCATTGCCGCGTCTTTTGCGGAAGGCGAAACCGT

GATGGACGGTCTCGATGAACTGCGCGTCAAGGAATCGGATCGTCTGGCGGCCGTTGCGCG

CGGCCTTGAAGCCAATGGTGTCGATTGTACCGAAGGCGAGATGTCGCTGACGGTTCGTGG

CCGCCCCGGCGGCAAGGGGCTGGGCGGTGGCACGGTTGCAACCCACCTCGACCACCGCAT

CGCGATGAGTTTCCTCGTCATGGGCCTTGCATCGGAAAAGCCGGTTACGGTGGATGACAG

CACCATGATCGCCACCTCTTTCCCG

>glk_4

AAACCGGAGGAGGCTGTTGCCACCCGCGTCGTGCTCGGCCCCGGCACGGGCCTTGGCGTG

GCAGGTCTGGTTCGCACACGTCATGCATGGGTTCCGGTTCCCGGTGAAGGCGGTCATATC

GATATCGGTCCACGCACCGAACGCGACTACCAGATTTTCCCGCATATCGAACGCATCGAA

GGGCGTGTCACCGGCGAGCAAATTCTTAGCGGGCGGGGCCTGCGCAACCTCTATCTGGGC

ATCTGCGCCGCCGACAAGATCACGCCCACCCTTGAGACGCCAGTAGACATTACATCCGCC

GGACTGGACGGCAGCAATCCACAAGCCGCAGAAACGCTTGACCTCTTCGCCACCTATCTG

GGGCGGCTTGCGGGCGACCTTGCGCTCATTTTCATGGCGCATGGCGGCGTTTATCTTTCG

GGTGGCATCCCGGTGCGCATCCTTTCCGCCCTCAAGGCCGGTTCGTTCCGCGCAG

>dnaK_2

TTGCCGAGTTCAAGAAGGAAAGTGGCATCGACCTGAAGAACGACAAGCTTGCCCTGCAGC

GCCTCAAGGAAGCTGCCGAAAAGGCCAAGATCGAACTGTCGTCCTCGCAGCAGACCGAAA

TCAACCTGCCGTTCATCACGGCTGACCAGACTGGCCCGAAGCATCTGGCGATCAAGCTGT

CGCGCGCCAAGTTTGAAAGCCTGGTCGATGATCTCGTGCAGCGCACGGTCGAGCCGTGCA

AGGCGGCGCTCAAGGATGCCGGCCTCAAGGCTGGCGAAATTGACGAAGTGGTTCTGGTCG

GCGGCATGACCCGCATGCCCAAGATTCAGGAAGTCGTGAAGGCCTTCTTCGGCAAGGAAC

CGCACAAGGGCGTGAACCCGGATGAAGTCGTGGCCATGGGCGCGGCGATCCAGGGCGGCG

TTTTGCAGGGCGACGTCAAGGACGTGCTGCTGCTCGACGTGACCCCGCTT

>gyrB_1

GGGCACCCTCATCACGGCGCTTGGCACCTCCATCGGCAAGGATGAAACGCACGGCTTCAA

CGCCGACAAGCTGCGTTATCACAAGATCATCATCATGACCGACGCCGACGTCGATGGCGC

CCATATTCGTACGCTTCTGCTCACCTTCTTCTTCCGGCAGATGCCGGAACTGATCGAACG

CGGGCATATCTATATCGCGCAGCCGCCGCTCTATAAGGTGACACGCGGCAAGTCTTCGCA

ATATATCAAGAACGAAGCCGCCTTTGAGGATTTCCTCATCGAAACCGGCCTTGAAGAAAC

GACACTGGAACTGGTGACTGGCGAAATGCGCGCCGGGCCGGATTTGCGCTCGGTGGTGGA

GGATGCGCGCACGCTGCGTCAGCTTCTGCACGGCCTGCACACCCGCTATGACCGCAGCGT

GGTGGAACAGGCGGCAATTGCCGGCCTGCTCAACCCCGATGCCTCAAGG

>trpE_3

ATGCATTTCAATGGCGATATGAATACAGGGCTGACGCTGCGCACCATCCGCATCAAGGAT

GGTGTGGCGGAAATCCGTGCAGGGGCGACGCTTCTGTTCGATTCCAACCCTGACGAGGAA

GAAGCCGAGACCGAATTGAAGGCATCGGCCATGATTGCGGCTGTGCGGGACGCACAGAAG

AGCAATCAGATCGCGGAAGAAAGTGTGGCGGCAAAGGTGGGTGAGGGGGTTTCGATCCTG

CTGGTCGATCACGAGGATTCCTTCGTCCATACGCTTGCCAATTATTTCCGCCAGACGGGC

GCCAAGGTTTCCACCGTGCGTTCACCGGTGGCAGAGGAGATATTCGACCGCGTCAATCCC

GATCTGGTGGTGTTATCGCCGGGACCGGGCTCGCCGCAGGATTTCGATTGCAAGGCGACC

ATCGATAAGGCGCGCAAGCGCCAGCTTCCGATTTTTGGCGTCTGCCTCGGCCTTCAGGCA

CTGGCG

>cobQ_1

CGGCGGATCAGTGACCCGGCGGGTATTGAAGGCAATGTGCGCGATATCGAGGGGCTGGGC

CTTCTCGATATCGAGACGATGACGGAGCCGGAAAAAGTGGTTCGCAATGTTGAGGCGGTG

TCGCTGCTGCATGATGAGCCGCTGGAGGGCTATGAAATCCACATCGGGCGCACCAGCGGG

CCGGATATGGCGCGGCCATTTGCGCGTATCGGCGATCATGATGATGGGGCCGTCTCGCCC

GATGGTCGTATCATGGGAACCTATCTCCACGGTATTTTCAGTGCGGATCGTTTCCGCCAC

CACTTTTTGCGCGCGCTGGGTGTGGAAGGCGGCCAGATGAATTATCGCGAGAGCGTCGAA

GAGGCTCTGGGCGAACTGGCTGAAGGGCTGGAAGCCTCGCTGGATATTGATGGCCTGTTT

GC

>omp25_1

TCTCGTAATCGTCTCGGCTGCGCTGCTGCCGTTCTCTGCGACCGCTTTTGCTGCCGACGC

CATCCAGGAACAGCCTCCGGTTCCGGCTCCGGTTGAAGTAGCTCCCCAGTATAGCTGGGC

TGGTGGCTATACCGGTCTTTACCTTGGCTATGGCTGGAACAAGGCCAAGACCAGCACCGT

TGGCAGCATCAAGCCTGACGATTGGAAGGCTGGCGCCTTTGCTGGCTGGAACTTCCAGCA

GGACCAGATCGTATACGGTGTTGAAGGTGATGCAGGTTATTCCTGGGCCAAGAAGTCCAA

GGACGGCCTGGAAGTCAAGCAGGGCTTTGAAGGCTCGCTGCGTGCCCGCGTCGGCTACGA

CCTGAACCCGGTTATGCCGTACCTCACGGCTGGTATTGCCGGTTCGCAGATCAAGCTTAA

CAACGGCTTGGACGACGAAAGCAAGTTCCGCGTGGGTTGGACGGCTGGTGCCGGTCTCGA

AGCCAAGCTG

>int_hyp_4

TAACGGTGGTATCATACCGAGTAAAAGAGCGATCTTTACACCCTTGTCGATCCTGTTTCG

CCCCCGCCACAACACAGCCTGATCGGCAAGCTGTGCTGTGGTGGAGGCGCCGGGTACCGC

CCCCGGGTCCAATGGGTTTATTACACCGTCCGTTTATCACCATAGTCGGCTTGCGCCGAC

AGGACGTATATAGGCGTGGTTTTTACCGATTGGAAGGGGGCTTGTGCGTTTTCGCGCAAG

ACCGACAGAGGTGGTGCGGCCCTTCCGTTCATTTTCCATTGACAGCTTCCGCGTGCTGGT

CAATCCTCACAATATATCGGGATCGGCCTTGAAGAGGCTTGGCGCAGCCGGGGCGGAAAC

CATGGCTGAAACGGGGACGATATGCCCCAATCGAAGGAGAGTGGATATATGAGTGAATAT

CTCGCGGATG

ST83

>gap_1

CTCCGCTCCCGCAGACGGTGCCGATCTCACCGTCGTCTATGGTGTCAACAACGACAAGCT

GACGAAGGACCATCTGGTCATCTCCAACGCTTCGTGCACCACCAACTGCCTTGCGCCGGT

GGCTCAGGTTCTCAACGATACTATCGGTATCGAAAAGGGCTTCATGACCACGATCCACTC

CTATACGGGCGACCAGCCGACGCTGGACACCATGCACAAGGATCTCTACCGCGCCCGCGC

CGCTGCCCTTTCCATGATCCCGACCTCGACGGGTGCGGCCAAGGCCGTCGGTCTCGTTCT

GCCGGAACTGAAAGGCAAGCTCGACGGCGTTGCCATTCGCGTCCCGACCCCAAATGTCTC

GGTCGTTGATCTCACCTTCATCGCCAAGCGTGAAACCACCGTTGAAGAAGTCAACAATGC

GATCCGCGAAGCCGCCAATGGCCGCCTCAAGGGCATTCTCGGCTATACCGATGAGAAGCT

CGTCTCGCACGACTTCAACCACGATTCCCATTCCTCGGTCTTCCACACCGACCAGACCAA

GGTTATGGACGGCACCATGGTGCGTATCCTGTCGTGGTACGACAATGAA

>aroA_6

TGATCCCTCGTCAACGGCTTTTCCGCTGGTGGCCGCCCTTCTGGTCGAAGGTTCGGACGT

CACCATCCGCAATGTGCTGATGAACCCGACCCGCACCGGCCTGATCCTGACGTTGCAGGA

AATGGGGGCGGATATCGAGATCATCGATCCACGCCTTGCCGGCGGCGAGGATGTCGCCGA

TCTGCGCGTCAGGGCCTCGAAGCTGAAAGGCGTTGTCGTTCCGCCGGAACGTGCGCCTTC

GATGATCGATGAATATCCGGTTCTGGCCATTGCCGCGTCTTTTGCGGAAGGCGAAACCGT

GATGGACGGTCTCGATGAACTGCGCGTCAAGGAATCGGATCGTCTGGCGGCCGTTGCGCG

CGGCCTTGAAGCCAATGGTGTCGATTGTACCGAAGGCGAGATGTCGCTGACGGTTCGTGG

CCGCCCCGGCGGCAAGGGGCTGGGCGGTGGCACGGTTGCAACCCACCTCGACCACCGCAT

CGCGATGAGTTTCCTCGTCATGGGCCTTGCATCGGAAAAGCCGGTTACGGTGGATGACAG

CACCATGATCGCCACCTCTTTCCCA

>glk_4

AAACCGGAGGAGGCTGTTGCCACCCGCGTCGTGCTCGGCCCCGGCACGGGCCTTGGCGTG

GCAGGTCTGGTTCGCACACGTCATGCATGGGTTCCGGTTCCCGGTGAAGGCGGTCATATC

GATATCGGTCCACGCACCGAACGCGACTACCAGATTTTCCCGCATATCGAACGCATCGAA

GGGCGTGTCACCGGCGAGCAAATTCTTAGCGGGCGGGGCCTGCGCAACCTCTATCTGGGC

ATCTGCGCCGCCGACAAGATCACGCCCACCCTTGAGACGCCAGTAGACATTACATCCGCC

GGACTGGACGGCAGCAATCCACAAGCCGCAGAAACGCTTGACCTCTTCGCCACCTATCTG

GGGCGGCTTGCGGGCGACCTTGCGCTCATTTTCATGGCGCATGGCGGCGTTTATCTTTCG

GGTGGCATCCCGGTGCGCATCCTTTCCGCCCTCAAGGCCGGTTCGTTCCGCGCAG

>dnaK_1

TTGCCGAGTTCAAGAAGGAAAGTGGCATCGACCTGAAGAACGACAAGCTTGCCCTGCAGC

GCCTCAAGGAAGCTGCCGAAAAGGCCAAGATCGAACTGTCGTCCTCGCAGCAGACCGAAA

TCAACCTGCCGTTCATCACGGCTGACCAGACTGGCCCGAAGCATCTGGCGATCAAGCTGT

CGCGCGCCAAGTTTGAAAGCCTGGTCGATGATCTCGTGCAGCGCACGGTCGAGCCGTGCA

AGGCGGCGCTCAAGGATGCCGGCCTCAAGGCTGGCGAAATTGACGAAGTGGTTCTGGTTG

GCGGCATGACCCGCATGCCCAAGATTCAGGAAGTCGTGAAGGCCTTCTTCGGCAAGGAAC

CGCACAAGGGCGTGAACCCGGATGAAGTCGTGGCCATGGGCGCGGCGATCCAGGGCGGCG

TTTTGCAGGGCGACGTCAAGGACGTGCTGCTGCTCGACGTGACCCCGCTT

>gyrB_4

GGGCACCCTCATCACGGCGCTTGGCACCTCCATCGGCAAGGATGAAACGCACGGCTTCAA

CGCCGACAAACTGCGTTATCACAAGATCATCATCATGACCGACGCCGACGTCGATGGCGC

CCATATTCGTACGCTTCTGCTCACCTTCTTCTTCCGGCAGATGCCGGAACTGATCGAACG

CGGGCATATCTATATCGCGCAGCCGCCGCTCTATAAGGTGACACGCGGCAAGTCTTCGCA

ATATATCAAGAACGAAGCCGCCTTTGAGGATTTCCTCATCGAAACCGGCCTTGAAGAAAC

GACACTGGAACTGGTGACTGGCGAAATGCGCGCCGGGCCGGATTTGCGCTCGGTGGTGGA

GGATGCGCGCATGCTGCGTCAGCTTCTGCACGGCCTGCACACCCGCTATGACCGCAGCGT

GGTGGAACAGGCGGCAATTGCCGGCCTGCTCAACCCCGATGCCTCAAGG

>'trpE-13'

ATGCATTTCAATGGCGATATGAATACAGGGCTGACGCTGCGCACCATCCGCATCAAGGATGGTGTGGCGGAAATCCGTGCAGGGGCGACGCTTCTGTTCGATTCCAACCCTGACGAGGAAGAAGCCGAGACCGAATTGAAGGCATCGGCCATGATTGCGGCTGTGCGGGACGCACAGAAGAGCAATCAGATCGCGGAAGAAAGTGTGGCGGCAAAGGTGGGTGAGGGGGTTTCGATCCTGCTGGTCGATCACGAGGATTCCTTCGTCCATACGCTTGCCAATTATTTCCGCCAGACGGGCGCCAAGGTTTCCACCGTGCGTTCACCGGTGGCAGAGGAGATATTCGACCGCGTCAATCCCGATCTGGTGGTGTTATCGCCGGGACCGGGCTCGCCGCAGGATTTCGATTGCAAGGCGACCATCGATAAGGCGCGCAAGCGCCAGCTTCCGATTTTTGGCGTCTGCCTCGGCCTTGAGGGACTGGCG

>cobQ_5

CGGCGGATCAGTGACCCGGCGGGTATTGAAGGCAATGTGCGCGATATCGAGGGGCTGGGC

CTTCTCGATATCGAGACGATGATGGAGCCGGAAAAAGTGGTTCGCAATGTTGAGGCGGTG

TCGCTGCTGCATGATGAGCCGCTGGAGGGCTATGAAATCCACATCGGGCGCACCAGCGGG

CCGGATATGGCGCGGCCATTTGCGCGTATCGGCGATCATGATGATGGGGCCGTCTCGCCC

GATGGTCGTATCATGGGAACCTATCTCCACGGTGTTTTCAGTGCGGATCGTTTCCGCCAC

CACTTTTTGCGCGCGCTGGGTGTGGAAGGCGGCCAGATGAATTATCGCGAGAGCGTCGAA

GAGGCTCTGGACGAACTGGCTGAAGGGCTGGAAGCCTCGCTGGATATTGATGGCCTGTTT

GC

>omp25_2

TCTCGTAATCGTCTCGGCTGCGCTGCTGCCGTTCTCTGCGACCGCTTTTGCTGCCGACGC

CATCCAGGAACAGCCTCCGGTTCCGGCTCCGGTTGAAGTAGCTCCCCAGTATAGCTGGGC

TGGTGGCTATACCGGTCTTTACCTTGGCTACGGCTGGAACAAGGCCAAGACCAGCACCGT

TGGCAGCATCAAGCCTGACGATTGGAAGGCTGGCGCCTTTGCTGGCTGGAACTTCCAGCA

GGACCAGATCGTATACGGTGTTGAAGGTGATGCAGGTTATTCCTGGGCCAAGAAGTCCAA

GGACGGCCTGGAAGTCAAGCAGGGCTTTGAAGGCTCGCTGCGTGCCCGCGTCGGCTACGA

CCTGAACCCGGTTATGCCGTACCTCACGGCTGGTATTGCCGGTTCGCAGATCAAGCTTAA

CAACGGCTTGGACGACGAAAGCAAGTTCCGCGTGGGTTGGACGGCTGGTGCCGGTCTCGA

AGCCAAGCTG

>int_hyp_1

TAACGGTGGTATCATGCCGAGTAAAAGAGCGATCTTTACACCCTTGTCGATCCTGTTTCG

CCCCCGCCACAACACAGCCTGATCGGCAAGCTGTGCTGTGGTGGAGGCGCCGGGTACCGC

CCCCGGGTCCAATGGGTTTATTACACCGTCCGTTTATCACCATAGTCGGCTTGCGCCGAC

AGGACGTATATAGGCGTGGTTTTTACCGATTGGAAGGGGGCTTGTGCGTTTTCGCGCAAG

ACCGACAGAGGTGGTGCGGCCCTTCCGTTCATTTTCCATTGACAGCTTCCGCGTGCTGGT

CAATCCTCACAATATATCGGGATCGGCCTTGAAGAGGCTTGGCGCAGCCGGGGCGGAAAC

CATGGCTGAAACGGGGACGATATGCCCCAATCGAAGGAGAGTGGATATATGAGTGAATAT

CTCGCGGATG
