# Supplementary figures and images for: Genetic polymorphisms identify in species/biovars of Brucella isolated in China between 1953 and 2013 by MLST
Source: BMC Microbiol. 2018 Jan 10;18:7. doi: 10.1186/s12866-018-1149-0 (PMC5781281; doi:10.1186/s12866-018-1149-0)

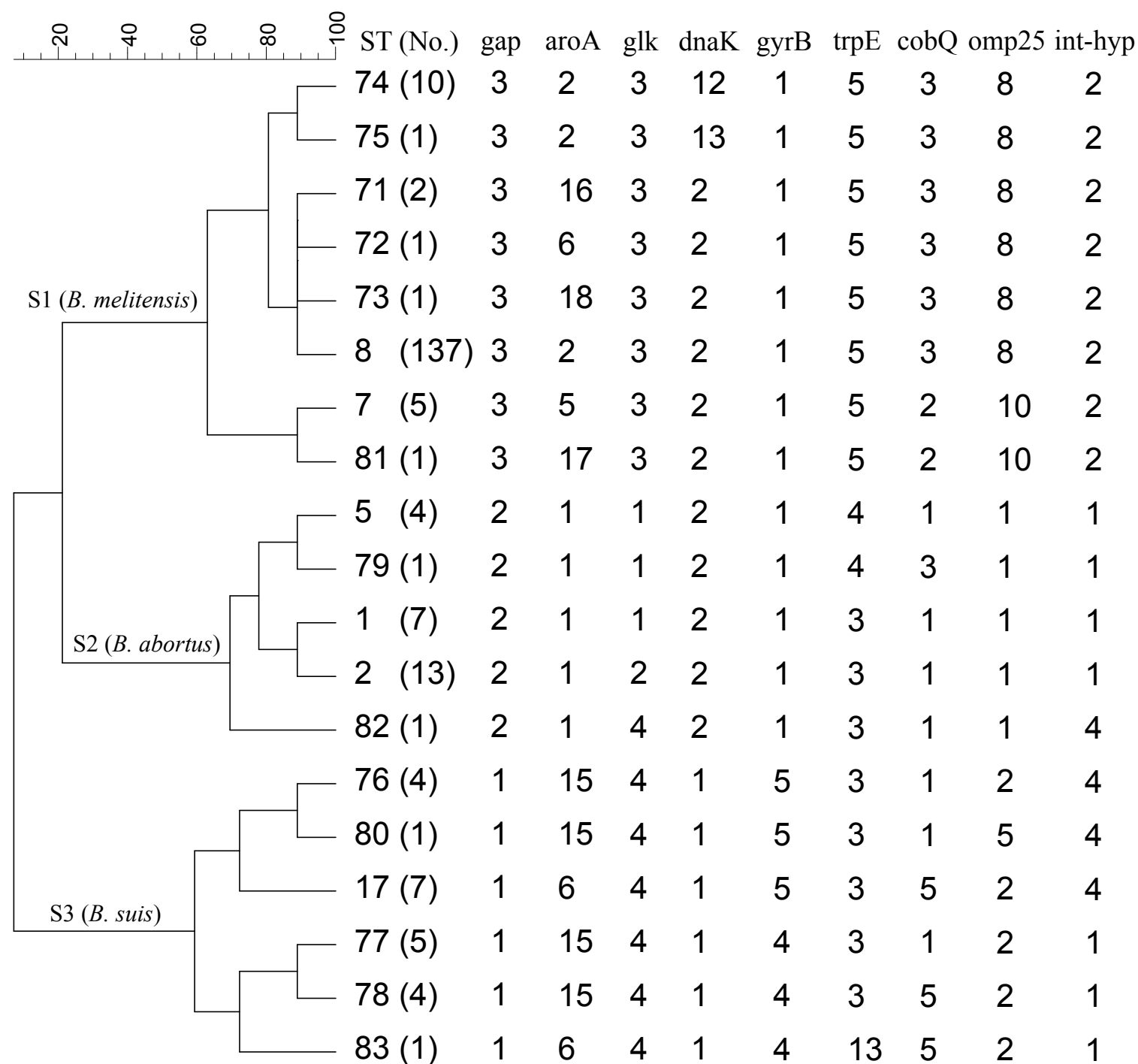

Supplement: Supplementary file 3 — UPGMA dendrogram based on the MLST assay showing the similarities of 206 Brucella isolates. Key: serial number for the 206 isolates; Biovar: stains species and biovars by phenotype; Place/Time: the place and time when the strains were collected; Source: the hosts from which the bacteria was isolated; ST: MLST genotype. (PDF 221 kb) [file 12866_2018_1149_MOESM3_ESM.pdf]
